# Tuning PFKFB3 Bisphosphatase Activity Through Allosteric Interference

**DOI:** 10.1038/s41598-019-56708-0

**Published:** 2019-12-30

**Authors:** Helena Macut, Xiao Hu, Delia Tarantino, Ettore Gilardoni, Francesca Clerici, Luca Regazzoni, Alessandro Contini, Sara Pellegrino, Maria Luisa Gelmi

**Affiliations:** 1 DISFARM- Department of Pharmaceutical sciences, Via Mangiagalli 25, 20133 Milan, Italy; 2 Department of Biosciences, University of Milan, Via Celoria 26, 20133 Milan, Italy

**Keywords:** Biochemistry, Chemical biology

## Abstract

The human inducible phospho-fructokinase bisphosphatase isoform 3, PFKFB3, is a crucial regulatory node in the cellular metabolism. The enzyme is an important modulator regulating the intracellular fructose-2,6-bisphosphate level. PFKFB3 is a bifunctional enzyme with an exceptionally high kinase to phosphatase ratio around 740:1. Its kinase activity can be directly inhibited by small molecules acting directly on the kinase active site. On the other hand, here we propose an innovative and indirect strategy for the modulation of PFKFB3 activity, achieved through allosteric bisphosphatase activation. A library of small peptides targeting an allosteric site was discovered and synthesized. The binding affinity was evaluated by microscale thermophoresis (MST). Furthermore, a LC-MS/MS analytical method for assessing the bisphosphatase activity of PFKFB3 was developed. The new method was applied for measuring the activation on bisphosphatase activity with the PFKFB3-binding peptides. The molecular mechanical connection between the newly discovered allosteric site to the bisphosphatase activity was also investigated using both experimental and computational methods.

## Introduction

Phospho-fructokinase bisphosphatases (PFKFBs) are bifunctional enzymes possessing both kinase and phosphatase sites, and are important in cell metabolism. These enzymes catalyse the forward and reverse reactions of converting fructose-6-phosphate to fructose-2,6-bisphosphate (F2,6BP)^1–3^, regulating intracellular F2,6BP level. F2,6BP is a strong allosteric activator of 6-phosphofructo-1-kinase (PFK-1) and a potent stimulator of glycolysis^4,5^. 6-Phosphofructo-2-kinase/fructose-2,6-biphosphatase 3 (PFKFB3) is the isoform 3 of the PFKFBs family, also known as the inducible isoform. Among all the isoforms, PFKFB3 was demonstrated with an extraordinarily high kinase to phosphatase activities ratio (around 740:1)^6^. Furthermore, PFKFB3 was determined to play an important role in the Warburg effect and the hypoxia response^7–13^ and has become a worthy target for modulating abnormal cellular metabolic dysfunctions, such as in cancer and atherosclerosis.

As a consequence, studies intended for PFKFB3 kinase inhibition have been ongoing since early 2000s and low-μM to low-nM kinase inhibitors have been discovered^14–21^. Although decent PFKFB3 kinase inhibition was achieved, the limitations and off-target issues related to ATP competitive inhibition still hinder further developments of the kinase inhibitors. Therefore, an indirect approach through phosphatase activation could represent an unexplored alternative towards PFKFB3 activity modulation. PFKFB3 bisphosphatase activity is controlled by the *N*-terminal autoregulatory domain that forms a *β*-hairpin structure as observed in crystal^22^. Its interaction with a large portion of the phosphatase region could result in a structural perturbation of the protein, strengthening the binding of fructose-6-phosphate to the active site and *de facto* inhibiting the release of the phosphatase product^22^. In the liver isoform, which contains a *N*-terminus structure similar to that of PFKFB3, the deletion of this autoregulatory domain led to an increase in the bisphosphatase/kinase activity ratio up to 12 folds compared to the wild-type enzyme^23^. Hence, it is likely that molecules perturbing the *β*-hairpin interaction with the PFKFB3 phosphatase domain could lead to phosphatase activation. Nevertheless, to our knowledge, the allosteric activation of PFKFB3 bisphosphatase by means of ligand interference has never been attempted before.

In this study, we applied virtual screening (VS) to discover potential allosteric PFKFB3 bisphosphatase activators. The binding affinity of the selected compounds was evaluated by microscale thermophoresis (MST), while a LC-MS/MS analytical method was developed for a direct evaluation of PFKFB3 bisphosphatase activity. Overall, we identified three compounds able to bind PFKFB3 and two of them significantly increased its bisphosphatase activity, without affecting the kinase activity. Molecular dynamic (MD) simulations were also performed on different states of PFKFB3 (unbound, substrate-bound, and product-bound). The analysis of the MD trajectories provided further insights on the interactions between the allosteric site and the two functional domains, which could play a role in the fine regulation of the kinase/phosphatase activity.

## Materials and Methods

### PFKFB3 protein structure for VS

The PFKFB3 structure with PDB ID 2i1v^24^ was chosen as the starting structure. It provided a reasonable resolution at 2.50 Å, and the major part of the *β*-hairpin region was resolved. The complexed crystal has the natural enzymatic products bound at both binding sites and a *PISA*-constructed dimeric structure is also available from the PDB. Each monomer contains 449 amino acid residues. The MOE 2015 software package^25^ was used for the preparation of dimeric structures. All crystal water and phosphonic acid molecules were deleted. The missing loops (Pro28-Asn32 and Ser445-Asn453) of the crystal structure were constructed through a standard loop building procedure included in the “Structure Preparation” function of MOE^25^. Large section of the *C*-terminus (Val461-His520) is absent in the crystal structure, hence, was not considered in this study. This omission was considered reasonable in this study as the missing *C*-terminus is relatively far away from the sites of interests. Both *N*- and *C*-termini of the starting protein structure were capped to prevent terminal artefacts. All missing hydrogens are added using the “Protonate 3D” method provided in MOE.

### VS

Two screening libraries were derived from the Zinc database^26^ with the sizes of 644 and 4819 compounds, respectively. The libraries were manually picked from by-target ADME/T and Functional categories and any molecule targeting kinases was also excluded. The libraries were downloaded in the format of SMILES and 3D structures were constructed using MOE^27^. Each library was screened using both a *N*-terminal truncated PFKFB3 (PFKFB3.L2_P28del hereafter) and a full-length PFKFB3 (PFKFB3.full hereafter) structure. The size of the docking site is defined by considering the size of the largest molecule presented within the screened library, increasing the half value of its longest axis by 2 Å, and rounded up to the closest integer. The final binding site radius was 15 Å for both PFKFB3.L2_P28del and PFKFB3.full structures. Different ring conformations were generated and evaluated by SPORES included in the PLANTS docking software^28,29^. SDWASH^25^ was applied to determine reasonable tautomerism states of the ligand and stereoisomers were evaluated using SDSTEREO^25^. The structures were minimized briefly using the db_Minimize function within the MOE package. The docking of the generated configurations was performed using PLANTS^28,29^ with search speed set to 1. The ligands were ranked according to the score using the *PLANTS*_*chemplp*_ scoring method^28,29^. We used a score cut-off to select the better ranked ligands for further interaction analysis. The *PLANTS*_*chemplp*_ score cut-off of −100 was used. The compounds selected from each library for both PFKFB3.L2_P28del and PFKFB3.full structures were further filtered according to their interactions with the receptor. The interaction analyses were performed and visualized using the ligand interactions function included within the MOE package. Separate lists of testing compounds were generated for each library per receptor structure, and further experimental tests were performed as specified in later sections.

### MD simulations

Three states of PFKFB3 underwent MD simulations: the unbound, the substrate-bound, and the product-bound states. The initial crystal structure (PDB ID: 2i1v) already contained the natural enzymatic products within the binding site. The *apo* structure was generated by deleting the bound ligands of 2i1v. The substrate-bound state, on the other hand, was obtained by modifying the bound products by adding or deleting an additional phosphate group at the correct position. The modification was followed by a brief minimization *in situ* using the MOE package.

The foreign ligands (ATP, ADP, F2,6BP, and F6P) were re-parameterized for compatibility with AMBER simulation package^30^. The ATP and ADP parameters were published elsewhere^31^ and were downloaded from the AMBER parameter database^32^. The re-parameterizations of F2,6BP and F6P were performed using the RESP ESP charge Derive program (R.E.D.)^33^ and antechamber^34,35^. The R.E.D. program was applied to assign partial charges to atoms presented within F2,6BP and F6P. The structures of F2,6BP and F6P were first submitted to conformational search in MOE^26^ using the default settings. The two conformations with the lowest calculated energies were saved for partial charge assignment. For each conformer, two orientations were also adopted during the charging process by rotating the molecule by 180 degrees. The terminal oxygens within the phosphate groups in both F2,6BP and F6P were forced to be equivalently charged within each functional group. The atom types of *gaff* were assigned using antechamber.

The *ff14SB*^36^ and the *gaff*^34^ force fields were applied for PFKFB3 protein and the ligands, respectively. The topology and coordinate files required for MD simulations were generated using *tleap*^30^. The protein/complexes were firstly neutralized with sodium or chloride ions; 6 Cl^−^ were added for *apo* PFKFB3 and 10 Na^+^ were added for the product- and substrate-bound structures. The protein/complexes were then solvated with TIP3P explicit water molecules in a cubic box of 12 Å from protein surface to the boundaries. The neutralized and solvated structures were used as the starting structures for MD simulations.

MD simulations were run for *apo*, product-bound, and substrate-bound PFKFB3 structures in 3 replicates for each state. The *pmemd.MPI* module was used for all the simulations^30^. The energy minimization, heating, and equilibration procedures prior to MD simulations were all performed in multi-step manners, using the same procedure for every run. Non-bonded cut-off of 8 Å was applied throughout. The energy minimization started with relaxing hydrogen positions, with other atoms restrained, with 1000 steepest-decent (SD) steps followed by the conjugate gradient (CG) method to a maximum cycle of 5000. This step was followed by a 5000-max-cycle minimization (2000 SD + CG) of water and ions. The minimization was then extended onto side chains of amino acid for another 5000 maximum cycles (2500 SD + CG). The system then underwent 6 steps of heating to a final temperature of 300 K, under the NVT condition (constant volume and temperature with total number of atoms unchanged). From this step on, the SHAKE algorithm was applied to constraint bonds involving hydrogen atoms and Langevin dynamics was applied for temperature scaling. Each heating step was performed with a controlled heating of 50 K gap over 5 ps with a 0.0005 ps time step, while protein backbone was weakly restrained (10 kcal· mol^−1^·Å^−2^). After each 50 K heating process, the system was equilibrated for an additional 5 ps at the targeted temperature. After the temperature of the simulated system reached 300 K, a 200 ps equilibration was performed under NVT condition with the backbone weakly restrained (5 kcal· mol^−1^·Å^−2^). The simulation condition was then switched to NPT (constant pressure and temperature with total number of atoms unchanged) and equilibrated for another 200 ps with the 5 kcal· mol^−1^·Å^−2^ restraint still applied on protein backbone. Following this, 5 steps of 500-ps equilibration runs were performed to reduce the restraint weight on protein backbone gradually. Each step reduced the restraint by 1 kcal·mol^−1^·Å^−2^ until the restraint was completely removed. Lastly, MD simulations were performed under NPT condition at 300 K for 200 ns for each PFKFB3 state and repeats, with a time step set to 0.002 ps. Frames were recorded at a 1-ps frequency and trajectories were written in the binary NetCDF format. The trajectories were processed and analyzed using *cpptraj*^30^. The root-mean-squared deviations against time for all simulations is depicted in Figure S1, Supporting Information.

### Peptide synthesis

Amino acids, coupling reagents and cleavage chemicals were purchased from Sigma Aldrich and Fluorochem. Peptides were synthesized using manual solid phase peptide synthesis or automated microwave assisted solid phase synthesis on Wang or Rink amide resin using Fmoc protected amino acids and standard protocols^37^. Firstly, the *N*-Fmoc-protected amino acid was loaded on the resin (1.2 mmol/g loading). After *N*-deprotection with piperidine (20% in DMF), the following *N*-Fmoc-protected amino acid (5 eq.) was coupled to the resin using HOBT/HBTU (5 eq.) as activators and DIPEA (10 eq.) as the base. The Fmoc *N*-protecting group was removed, and the resin washed with DMF and DCM. Peptides were cleaved with a cleavage cocktail made of TFA (8 mL), water (200 µL), triisopropylsilane (400 µL), thioanisole (400 µL). The cleaved peptide was precipitated in cold diethylether and purified using the following RP-HPLC method: gradient elution of 5–70% solvent B (solvent A: water/acetonitrile/TFA, 95/5/0.1; solvent B: water/acetonitrile/TFA, 5/95/0.1) over 20 min at a flowrate of 20 ml/min using a C18 column.

### Protein expression

The PFKFB3 plasmid was provided by Prof. Yong-Hwan Lee from Louisiana State University. The His-tagged human inducible bifunctional enzyme (GenBank code: AF056320.1) was expressed in *Escherichia coli* BL21 (DE3) Plys as reported previously^22^. The PFKFB3 protein was purified using nickel affinity columns and the *N*-terminal His tag was removed using a standard protocol^24^. After the treatment with thrombin the protein was eluted with an anion-exchange column. The pure protein was concentrated to 2–10 mg/mL and stored in 20 mM Tris HCl pH 8.0, 10 mM NaPi, 5 mM β-mercaptoethanol and 5% glycerol.

### Binding assay

The dissociation constant was determined using Nanotemper microscale thermophoresis (MST)^38^. In a typical experiment, a compound was dissolved in MST buffer and mixed with 100 nM RED-NHS labeled recombinant PFKFB3 in 1:1 ratio. The mixture was incubated at room temperature for 20 min. and a full MST measurement was performed. Data were analyzed with Nanotemper software (https://nanotempertech.com/monolith/).

### Kinase assay

Promega ADP Glo kinase kit was purchased from Promega and a previously published protocol^18^ was used with modifications adjusted for this study. Kinase base buffer (pH 7.0) was prepared prior to the assay containing 100 mM HEPES, 400 mM KCl, 10 mM potassium phosphate, 10 mM MgCl_2_, 1 mM dithiothreitol, and 0.2 mM Triton X100. PFKFB3 enzyme was added to the base buffer immediately prior to starting the assay. Each well received 8 µL of enzyme diluted in the base buffer (final enzyme concentration in the assay was 150 nM). The tested compound was added to each well starting from 0.75 µM to 100 µM (8 concentrations). After 30 min incubation, each well received 8 µL of a mixture containing 100 µM ATP and 200 µM fructose-6-phosphate. The reactions were left to incubate for 1 h before the addition of 16 µL of ADP Glo reagent. After the second incubation of 50 min at room temperature, 32 µL of Kinase Detection Reagent was added. After an additional hour, the reaction mixtures were transferred to a white 384 well plate in triplicates. Tecan Magellan spectrometer with enhanced luminescence module was used to measure the luminescence signal generated in each well. If applicable, IC_50_ estimates were calculated using a commercially available software package from GraFit5.

### PFKFB3 phosphatase activity measurements

HPLC grade water (18 MΩ) was prepared with a Milli-Q purification system (Millipore, Milan, IT). Ammonium formate, fructose-6-phosphate (F6P), Tris HCl, glycerol, 2-mercaptoethanol, sodium phosphate and Amicon-ultra centrifugal filters (30 kDa) were purchased from Sigma Aldrich. F2,6BP was kindly provided by Prof. Lisardo Boscá from Spanish National Research Council in Madrid, Spain. Compound **AZ 33** was provided by Carlo DeDominicis, University of Aberdeen. Phosphatase activity of PFKFB3 was measured by quantitation of the reaction product (*i.e*. F6P) by means of liquid chromatography tandem mass spectrometry. Enzyme reaction rate was determined after 18 h of incubation at 37 °C in sterile (0.22 µm filtered) Tris buffer (20 mM Tris-HCl pH 8; 10 mM sodium phosphate; 5 mM 2-mercaptoethanol and 5% of glycerol) at a final enzyme concentration of 100 nM. Both the enzyme and the substrate (i.e. fructose-2,6-bisphosphate, F2,6BP) were pre-warmed at 37 °C for five min before starting the kinetics by spiking the substrate into the enzyme solution. To plot the enzyme kinetics, the reaction rate was measured in independent experiments at different concentration of substrate (i.e. 5, 10, 15, 20, 25, 30, 40 and 50 µM). Diafiltration on amicon-ultra filters (30 kDa MWCO) was used to stop the reactions. Both the residual substrate and the product were collected in the filtrate, while the enzyme was retained by the filter. The same protocol was used to measure the effect of ligands on enzyme phosphatase activity. For such a purpose the reaction rate was measured at the same substrate concentrations, but the enzyme was incubated with the compound and pre-warmed before starting the kinetics. Compounds were incubated with the enzyme at a concentration 50 µM or equal to their K_D_, if available. Independent analyses were done without the enzyme (i.e. controls) to assess any degradation of the substrate in the same experimental condition used for the enzyme kinetics. The concentration of the substrate produced by the kinetics was determined by interpolation with a calibration curve obtained by the analysis of fructose-6-phosphate standards prepared in a concentration range between 0.5 µM and 50 µM. The velocity was calculated as nanomoles of F6P produced in one min by a nanomole of PFKFB3, subtracting the nanomoles of F6P produced by self-degradation of the substrate from the nanomoles of F6P produced by the enzyme kinetic.

### Instrumental analysis

Quantitation of F6P produced was provided by a Surveyor HPLC system connected to a TSQ Quantum Ultra triple quadrupole mass spectrometer by a Finnigan IonMax electrospray ionization (ESI) source assembled with a high flow stainless steel emitter (Thermo Scientific, Rodano, MI, Italy). Filtrate containing the kinetics products were diluted 1:4 in water and placed into the autosampler rack, which was cooled at 8 °C to prevent sample degradation during analyses. Aliquots of 90 µL were injected and analyte separation was achieved at 40 °C by using Hypercarb column (100 × 2.1 mm; 5 µm particle size; 250 Å pore size, Thermo Scientific) at a flow rate of 300 µL/min. Table 1 reports the mobile phases and gradient program used for the elution.

**Table 1:** Gradient program for the elution and quantitation of fructose-6-phosphate by using Hypercarb column.

Time (s)	% H_2_O	% HCOO^−^ NH_4_^+^ 100 mM
0.00	100	0
1.00	100	0
1.01	60	40
4.00	60	40
4.01	100	0
8.00	100	0

The flow from the column was directly sprayed into the mass spectrometer with no further splitting. Nebulization and ionization were achieved by 70 units of sheath gas, 25 units of auxiliary gas, a capillary temperature of 270 °C and a spray voltage of −4.5 kV. The analyzer was set to scan the negative ions reported in Table 2 in Multiple Reaction Monitoring mode (MRM), with a scan time of 0.05 seconds and a resolution of 0.5 m/z for both the first and the third quadrupoles. The MRM transitions reported in Table 2 were previously optimized in a semi-automatic mode to achieve the best instrumental response and selectivity for fructose-6-phosphate, by flow injection of a standard solution.

**Table 2:** MRM transitions for selective determination of fructose-6-phosphate.

Precursor ion (m/z)	Fragment ion (m/z)	Collision energy (V)
258.92	79.1	45
258.92	97.1	20

## Results and Discussion

### VS

The objective of this work was to increase the bisphosphatase activity through allosteric intervention. The approach was initially inspired by the function of *N*-terminal autoregulatory domain, which could play a role in the bisphosphatase inhibition^22,39^. However, challenges remained as the dynamic of the *β*-hairpin structure in relation to its binding site was undetermined, and the intra-molecular interactions were only demonstrated in static crystal structures. The two symmetrical autoregulatory domains within a PFKFB3 dimer might well play a crucial role in stabilizing the dimeric structure. This could make a replacement or displacement of the autoregulatory domain a particularly complicated task to achieve. A secondary strategy was thus considered more plausible to ensure final success of PFKFB3 bisphosphatase activation.

**Figure 1:**
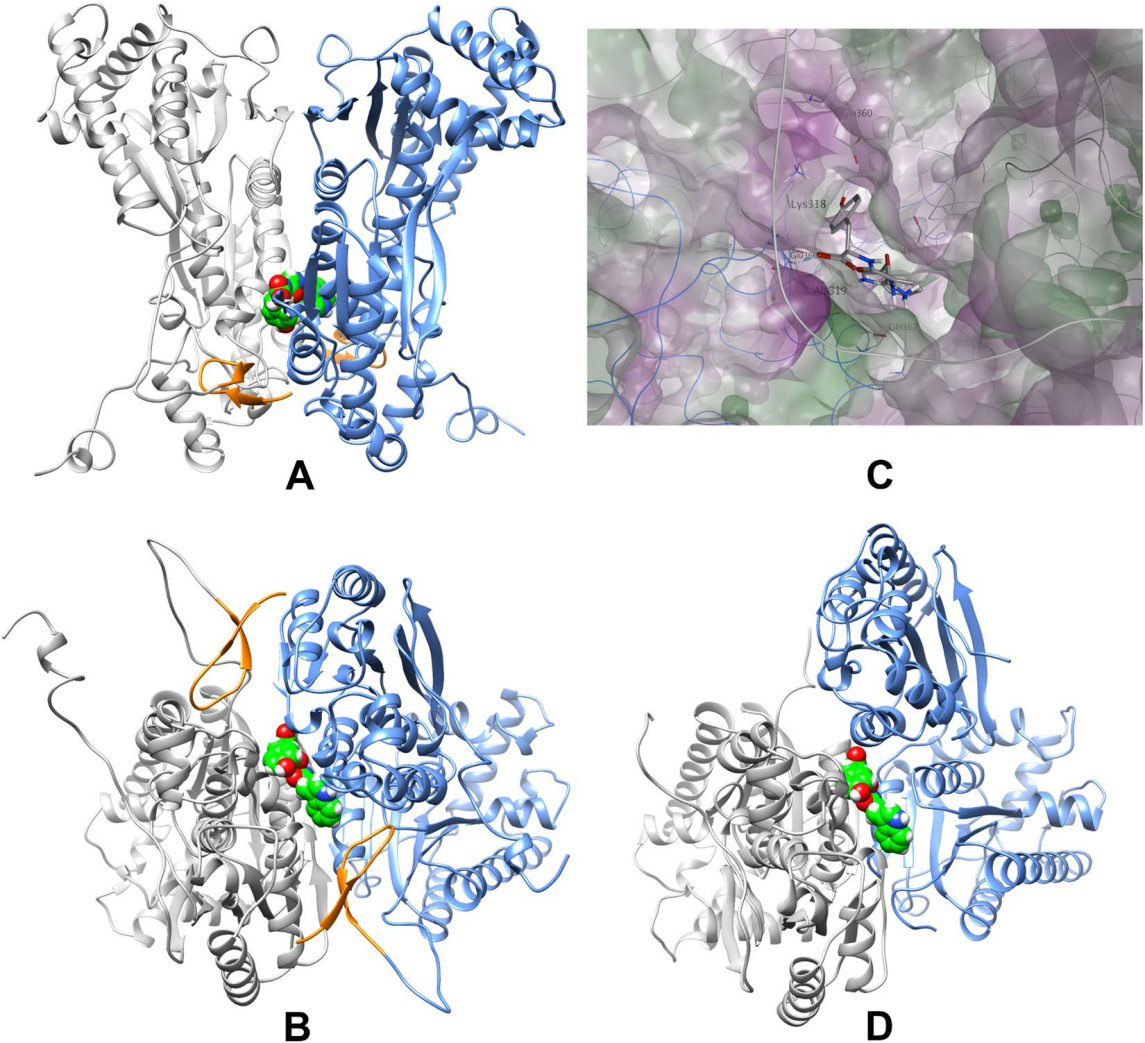
The relative positions of the two targeted sites in PFKFB3 and the structural comparison to PFKFB2. (**A**,**B**) The top-down (**A**) and sideway (**B**) representations of the N-terminal β-hairpin structure (orange) and the compound **6** (green) bound in the secondary binding site of PFKFB3. The two monomers are shown in different colors (grey and blue). (**C**) Surface representation of the secondary binding site with compound **6** docked within. The PFKFB3 surface is coloured according to residue lipophilicity (green: lipophilic; purple: hydrophilic). (**D**) Is the PFKFB2 structure with one monomer (white) overlaid with the PFKFB3 structure in B. The shifting of the monomer in blue due to the lack of stable β-hairpin can lead to the loss of the secondary binding site found in PFKFB3.

Some initial VS tests with the PFKFB3.L2_P28del form of the enzyme were performed. We noticed that most of the high ranked ligands bind preferably at a secondary site close to the *β*-hairpin binding site (Fig. 1A,B). This site is defined by Arg233, Gln299, Glu315-Ala319, Asn321, Asp324, Arg355, Glu360, Gln363-Arg368, and Glu370-Pro371 from both monomers. Further analysis and visual inspection suggested that this secondary binding site is in close vicinity to both the *β*-hairpin region and the bisphosphatase active site. The site provides a channel-like space to accommodate ligands (Fig. 1C) and is decorated by multiple charged and polar amino acid residues (e.g. Lys318, Glu360, Gln367, and Glu370). Moreover, the insertion of a ligand at this site might lead to a destabilization of the *β*-hairpin domain, or to a more direct interference with the bisphosphatase activity. It should be noted that this secondary binding site is highly conserved among PFKFB isozymes (83.3–88.9%). On the other hand, in the quaternary structure of PFKFB3 a clamping effect of the two N-terminus *β*-hairpins is observed. Thus, the differences at the N-terminus of other isoforms probably lead to a shift in the relative position of one monomer to another (Fig. 1B,D). This very likely alter the shape of the secondary binding site that can therefore be considered rather specific for PFKFB3.

Hence, two VS strategies were adopted in this study (strategy one and two, respectively). In strategy one, the ligand libraries used for VS were targeting at the *β*-hairpin domain binding site. The structure of the PFKFB3.L2_P28del form (*N*-terminus deleted) was used for this strategy. On the other hand, the PFKFB3.full structure was used for strategy two and ligand binding was targeted at the secondary binding site. The lists of ligands selected in VS for both strategies are shown in Tables S1 and S2, Supporting Information.

### Binding assay by MST

Peptides identified by VS were chemically synthesized using microwave assisted solid phase peptide synthesis (Table S3, Supporting Information). Their binding affinity toward PFKFB3 was assessed using Microscale Thermophoresis (MST). The most promising compounds that showed an evidence of binding, were tested at a wide concentration range. Key compounds are reported in Table 3. Three compounds exhibited a μΜ binding affinity toward the target of interest (compounds **4**–**6**). Of relevance, compounds **7** and **8** having the opposite stereochemistry of compounds **5** and **6**, were not able to bind the enzyme.

**Table 3:** Key compounds tested in this work.

Compound	MW	Binding Affinity (µM)
**4**	686,75	18 ± 1
**5**	633,80	44 ± 3
**6**	424,45	3 ± 1
**7**	747,87	No evidence of binding
**8**	424,45	No evidence of binding

**Figure 2:**
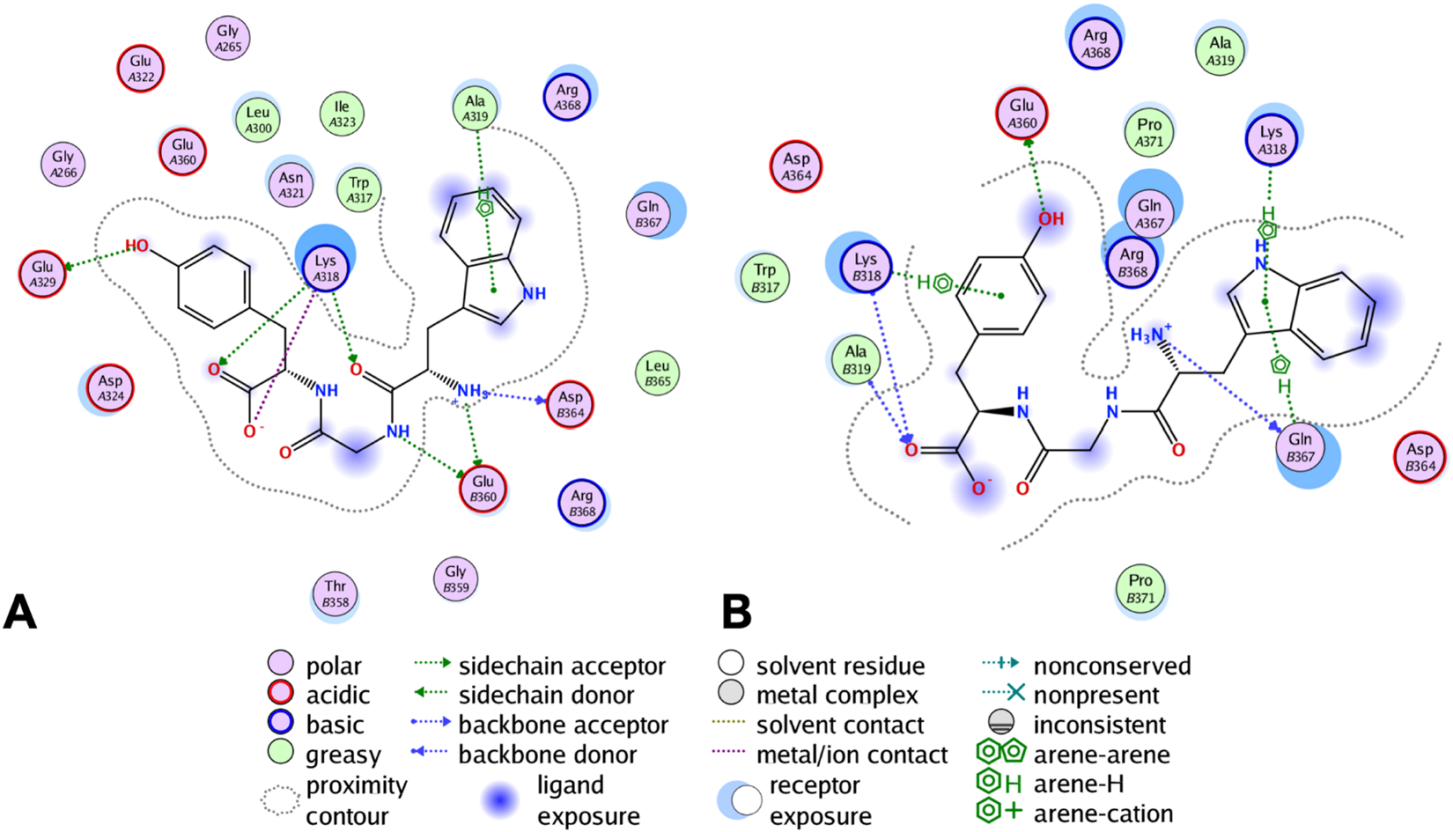
The ligand interactions of compound **8** and **6** binding with PFKFB3 in strategy one (**A**) and strategy two (**B**), respectively.

From the VS results, we noticed that compounds **4**, **5**, and **6** were selected by both strategies. However, visual inspection of the VS outcomes revealed that compound **8** was the selected stereoisomer at the binding site of strategy one (Fig. 2A), while compound **6** was preferred by the strategy two (Fig. 2B). Results from the experimental binding assay confirmed strategy two, where the full-length model of PFKFB3 was adopted as the most reliable since compound **8** was unable to bind PFKFB3. Moreover, the failure of strategy one confirms that the displacement of the autoregulatory domain may not be easily achieved by competitive ligands.

### Evaluating bisphosphatase activity by MS

**Figure 3:**
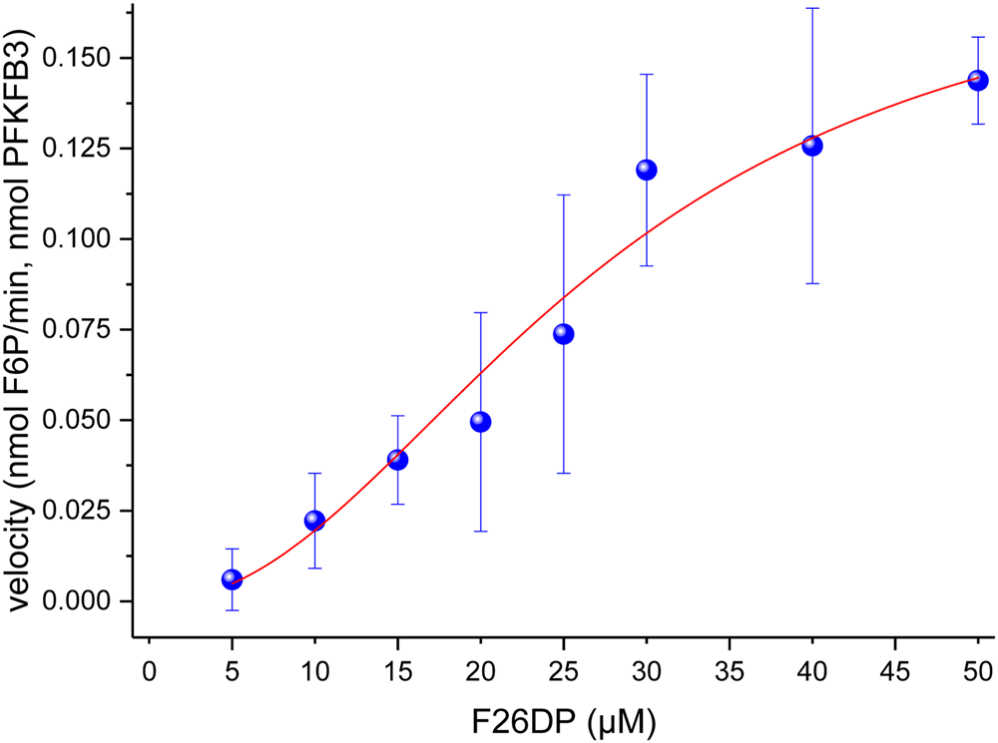
Bisphosphatase velocity (nmol/min of F6P per nmol of enzyme) as function of the substrate concentration (F26DP) for recombinant PFKFB3.

In order to determine the effect of the PFKFB3-binding compounds on the bisphosphatase activity, a LC-MS/MS analytical method was developed. Firstly, separate kinetics were measured for the enzyme alone or equilibrated with compounds **4–8**. The bisphosphatase activity of compound **AZ33**, a very potent PFKFB3 kinase inhibitor acting as a competitive binder of the ATP pocket^18^, was also evaluated, since it has not been reported before. Dunnett’s multiple comparisons test (two-way ANOVA) on the experimental results suggests that compounds **5**, **6** and **AZ33** significantly enhanced the bisphosphatase activity of the enzyme, whereas no compound has an opposite effect (see Table 4). The scatter plot of kinetics follows a sigmoidal trend (Fig. 3), therefore Hill Equation (1) was used for its fitting instead of a Michaelis-Menten equation:

1 $$V=\frac{{V}_{{\max }}\times {[S]}^{h}}{{{K}_{half}}^{h}+{[S]}^{h}}$$

where *V* is the kinetic velocity, *V*_*max*_ the maximum velocity at enzyme saturation, [S] the substrate concentration, *K*_*half*_ the substrate concentration giving a half of maximum velocity and *h* the Hill coefficient. The experimental parameters of the best fitting Hill equation are summarized in Table S4, which reports the values for the kinetics of the enzyme alone or equilibrated with compounds **4**–**8** and with the potent kinase inhibitor **AZ33**.

**Table 4:** Experimentally measured enzyme velocity at different substrate concentration.

Substrate	20 µM	25 µM	30 µM	40 µM	50 µM
**Enzyme velocity (nmol/min of F6P per nmol of enzyme)**					
PFKFB3	0.07 ± 0.01	0.09 ± 0.03	0.13 ± 0.01	0.13 ± 0.04	0.13 ± 0.04
PFKFB3 + 4	0.05 ± 0.03	0.07 ± 0.04	0.12 ± 0.03	0.13 ± 0.04	0.14 ± 0.01
PFKFB3 + 5	0.14 ± 0.03	0.17* ± 0.00	0.23** ± 0.02	0.28**** ± 0.07	0.36**** ± 0.00
PFKFB3 + 6	0.15** ± 0.02	0.17** ± 0.01	0.23**** ± 0.05	0.29**** ± 0.08	0.35**** ± 0.02
PFKFB3 + 7	0.07 ± 0.01	0.07 ± 0.04	0.11 ± 0.04	0.14 ± 0.01	0.14 ± 0.01
PFKFB3 + 8	0.06 ± 0.03	0.05 ± 0.00	0.10 ± 0.01	0.14 ± 0.05	0.14 ± 0.04
PFKFB3 + AZ33	0.15* ± 0.03	0.20*** ± 0.04	0.23** ± 0.04	0.25*** ± 0.04	0.36**** ± 0.01

Bold values for data significantly different from control (two-way ANOVA with Dunnett’s multiple comparisons test).

The observed sigmoidal trend (Fig. 3) suggests that PFKFB3 bisphosphatase has allosteric modulation sites. Specifically, a Hill coefficient value higher than one indicates a positive cooperativity between different sites or subunits^40,41^. The assay gave no false positives since no significant variations on Hill equation parameters or substrate conversion rate were observed after incubation with compounds **7** and **8**, which are unable to bind the enzyme as from MST assay (see Tables 4 and S4, Supporting Information). Moreover, the data reported in Table 4 prove that the enzyme binding is not enough for the modification of its bisphosphatase activity, since no significant variations on substrate conversion rate were observed upon incubation with compound **4**, which was previously identified as PFKFB3 binder by MST.

Interestingly, kinase inhibitor **AZ33** induces bisphosphatase activity modulation, as a significant increase of the experimentally measured substrate conversion rate was observed (see Table 4). Specifically, we observed an almost tenfold increase of the enzyme maximum velocity and a change of the kinetics from a sigmoidal to a Michaelis-Menten-like shape as from the Hill coefficient which decreased from 2 to 1.2 (see Table S4, Supporting Information). This observation implies that the occupation of the ATP binding site determines a loss of the enzyme cooperativity, followed by an allosteric activation of PFKFB3 bisphosphatase activity, which is otherwise auto-inhibited. It is rather intriguing that the compounds **5** and **6** gave quite superimposable kinetics modifications (see Tables 4 and S4, Supporting Information), although they were designed to bind sites far away from the ATP binding pocket. In addition, we tested the kinase activity of all peptides of interest and rewardingly, they resulted completely inactive. This demonstrates that PFKFB3 bisphosphatase activity could be conveniently stimulated without affecting the kinase.

### MD simulations

**Figure 4:**
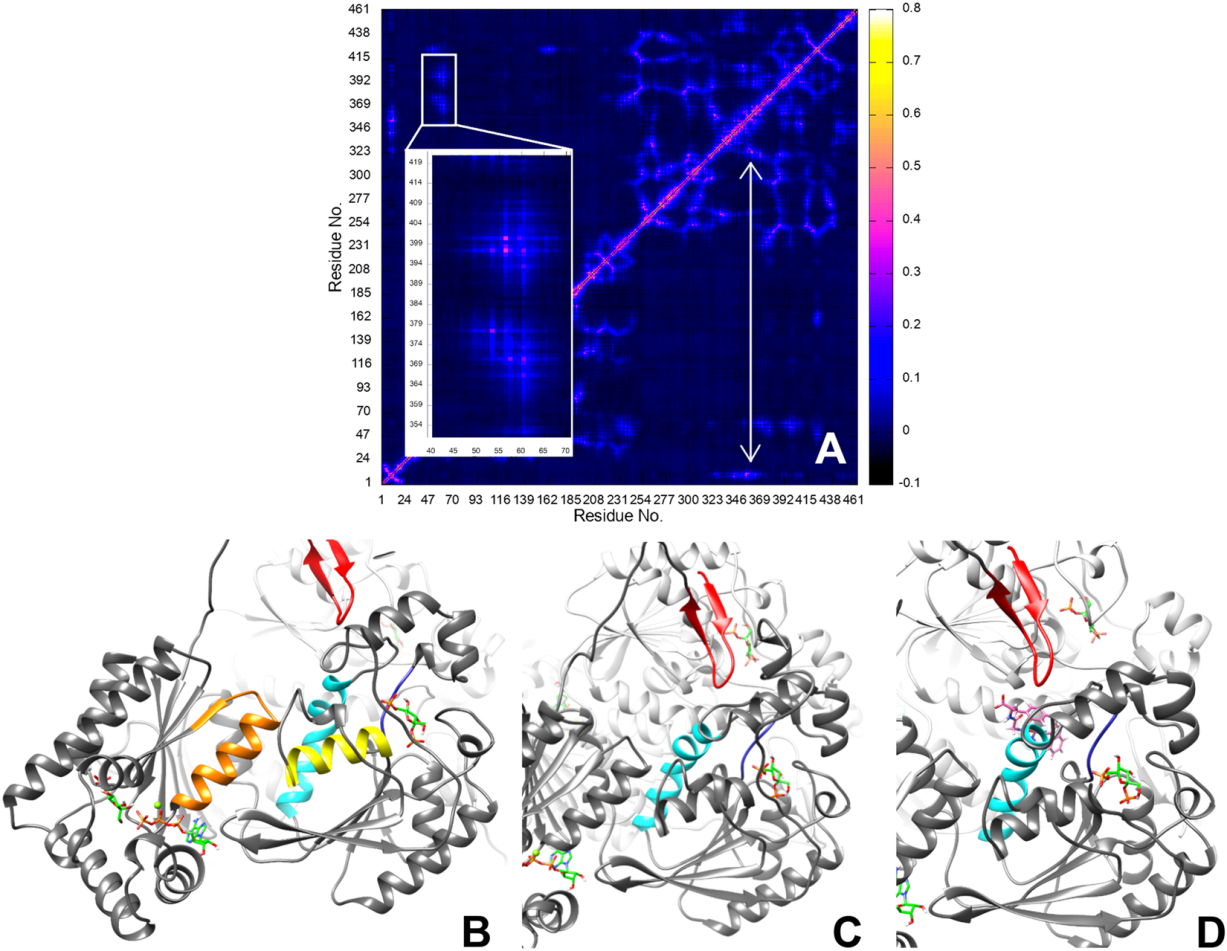
The atomic correlation heat map (**A**,**B**) by amino acid residues of MD simulation using apo PFKFB3, and the dynamically correlated regions under different scenario (**B**–**D**). (**A**) is the correlation heat map by residues of a PFKFB3 monomer of the apo structure. The box and the zoomed-in sub-plot show the three weakly correlated α-helices highlighted in (**B**) (α1 in orange, α17 in cyan, and α18 in yellow). The double-headed arrow in A shows the relatively stronger correlations of the autoregulatory domain (β-hairpin) to the Glu322-Ala325 loop (dark blue) through part of the α17-helix (Tyr362-Arg378, cyan). The β-hairpin (in red)-to-phosphatase correlated regions through α17 (cyan) are illustrated in (**C**). In (**D**), the docked pose of compound 6 (pink) indicates that the peptide is interfering with E322-A325 loop (dark blue) through the α17-helix (cyan). The enzyme substrates are all shown in green in (**B**–**D**).

MD simulations of three different states of PFKFB3 were performed in order to provide information on possible molecular mechanism of bisphosphatase modulation. All simulations have reached steady states within 100 ns according to backbone RMSD analysis (Figure S1, Supporting Information). Atomic correlation analysis of all the amino acid residues was performed on the last 100 ns of each MD simulation. Except the local dynamic correlation within kinase or bisphosphatase regions, we noticed a weak correlating zone that connects the ATP binding site to the bisphosphatase active site for all the simulated trajectories analyzed. (Fig. 4A) This occurs through the chained interactions between three α-helices – α1, α17, and α18. Moreover, α17 (Tyr362-Arg378) is the essential element to perturb the bisphosphatase activity. Indeed, part of the α17 helix lies parallel to the Glu322-Ala325 loop flooring the bisphosphatase site (Fig. 4B), to which is connected via Arg368 sidechain making a H-bond with Glu322 backbone carbonyl, as observed in the PFKFB3 X-ray structure^24^. More interestingly, the Glu322-Ala325 loop region is also indirectly connected to the N-terminal autoregulatory domain (Fig. 4C) that modulates bisphosphatase activity and is directly connected to the ATP binding loop (Pro43-Thr48)^24,39^. The indirect connection between the Glu322-Ala325 loop and the autoregulatory domain can also explain the experimental data showing similar results on the bisphosphatase activity for peptides **5** and **6** and **AZ-33**. However, while this latter compound was demonstrated to bind at the ATP binding loop and has kinase inhibiting activity^18^, compounds **5** and **6** might directly affect the conformation of the α17 helix, accordingly to their predicted binding mode (Figs. 2B and 4D), without affecting the kinase activity. Among the three ligands identified by VS and demonstrated as binders by MST, only compounds **5** and **6** showed phosphatase activation. Consequently, it is possible that compound **4** binds PFKFB3 on a different and non-functional binding site.

## Conclusions

Fructose 2,6-biphosphate is a potent activator of glycolysis. Its concentration is finely regulated by PFKFB3, a bifunctional enzyme constituted by a kinase domain (converting F6P to F2,6BP) and a bisphosphatase domain (converting F2,6BP to F6P). Due to its role in the cell cycle regulation, PFKFB3 raised the interest as a possible target for the treatment of pathologies related to abnormal cell metabolism. However, up to date, only strategies directed to the kinase domain have been reported by literature.

In this study, we report on the first attempt to achieve a modulation of PFKFB3 by activating the bisphosphatase. By combining molecular modelling and experimental techniques, we identified compounds **5** and **6** that are able to induce PFKFB3 bisphosphatase activation without affecting the kinase activity. Additionally, we observed that **AZ33**, a potent ATP competitive kinase inhibitor, is also able to modulate the bisphosphatase activity, although concurrently to kinase inhibition. Moreover, MD simulations conducted on unbound, substrate-bound and product-bound PFKFB3 showed that the motion of the ATP binding site and the bisphosphatase active site are weakly correlated. This correlation is mainly exerted through three dynamically connected α-helices (α1, α17, and α18) spanning from the ATP binding site to the Glu322-Ala325 loop that floors the bisphosphatase site. Thus, compounds **5** and **6** are possibly inducing bisphosphatase modulation by targeting the secondary binding site and through the direct interaction with the crucial α17 helix, that is in close contact with the Glu322-Ala325 loop. We are aware that a moderate increase in phosphatase activity, against a native 740:1 ratio in favour of the kinase, is unlikely to effectively deter cancer cell proliferation. Indeed, in this case, a more traditional approach based on kinase inhibition might still be preferred. However, allosteric phosphatase activation might be of interest to achieve a finer regulation of glycolysis without the risk of an off-target interaction with other kinases. Moreover, the compounds reported here can be valuable probes to study PFKFB3 under a so far unexplored condition with improved specificity. Furthermore, results reported here might be transferred to other PFKFB isoforms, opening the way to a potentially new approach for modern therapeutic opportunities.

## Supplementary information

Supplementary Information.
