# Supplementary material for: Tuning PFKFB3 Bisphosphatase Activity Through Allosteric Interference
Source: Sci Rep. 2019 Dec 30;9:20333. doi: 10.1038/s41598-019-56708-0 (PMC6937325; doi:10.1038/s41598-019-56708-0)

Tuning PFKFB3 Bisphosphatase Activity Through Allosteric Interference

Helena Macut[aγ], Xiao Hu[aγ], Delia Tarantino[b], Ettore Gilardoni[a], Francesca Clerici[a], Luca Regazzoni[a], Alessandro Contini[a]*, Sara Pellegrino[a]*and Maria Luisa Gelmi[a]

[a] DISFARM- Department of Pharmaceutical sciences, Via Mangiagalli 25, 20133, Milan, Italy.

[b] Department of Biosciences, University of Milan, Via Celoria 26, 20133, Milan, Italy.

[*] Corresponding authors: sara.pellegrino@unimi.it, alessandro.contini@unimi.it

[γ] Authors equally contributed to this work.

Supporting information

RMSD plot of MD simulations 2

Virtual screening results using strategy 1 3

Virtual screening results using strategy 2 5

List of synthesized peptides 12

Microscale thermophoresis results for binding peptides 13

Bisphosphatase assays data 15

Characterization of compounds 1-9 16

Figure S1: The RMSD plots of all 9 MD simulations performed.

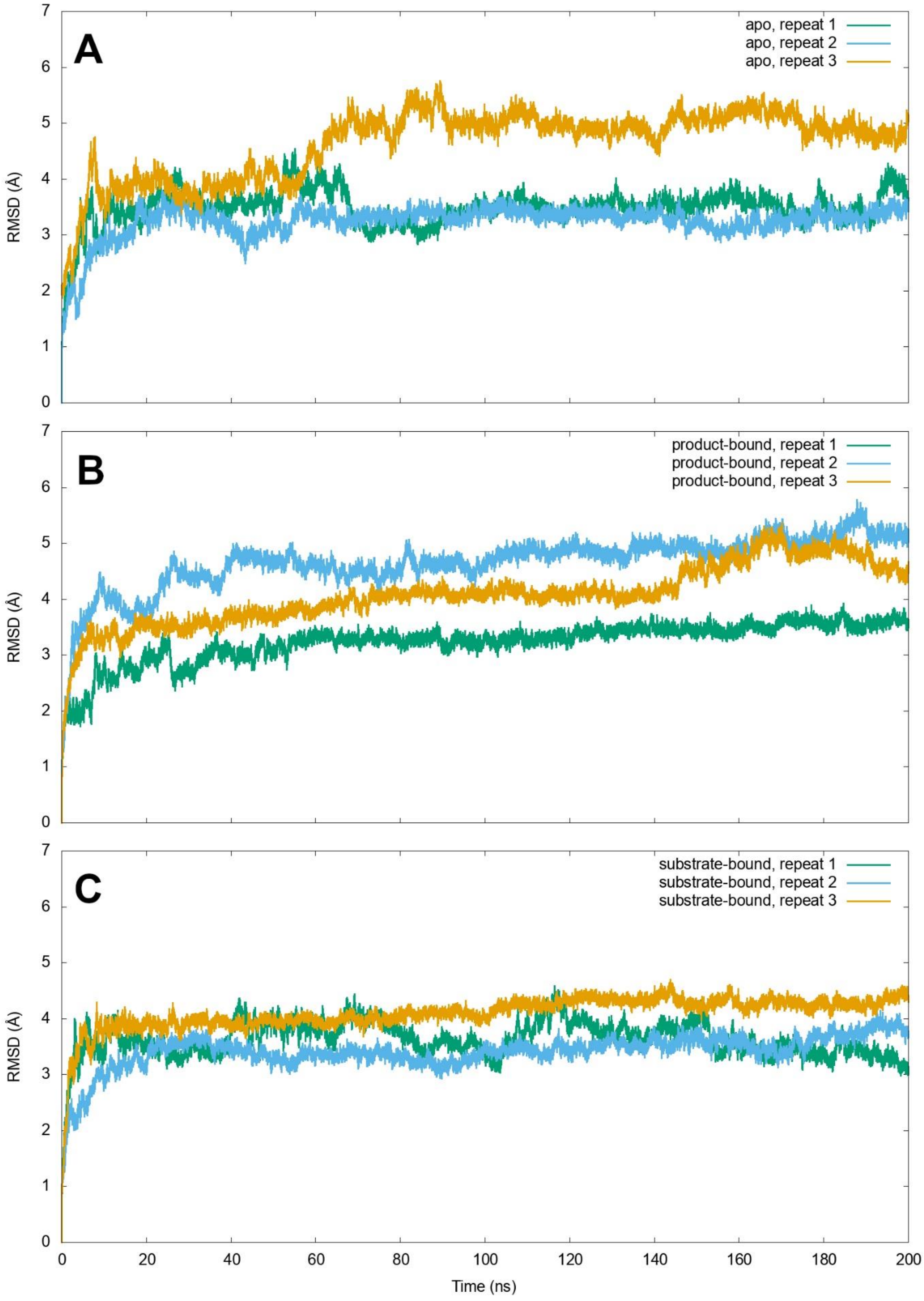

Table S1: Virtual screening results using strategy 1.

ZINC ID	VS score	Note	SMILES
ZINC03830635	-122.59		CC(=O)N(CCCCCNC(=O)CCC(=O)N(CCCCCNC(=O)CCC(=O)N(CCCCC[NH3+])O)O)O
ZINC29221575	-118.37		CC(C)C[C@@H](C(=O)N[C@@H](CC(C)C)C(=O)N[C@@H](CCCNC(=[NH2+])N)C(=O)N[C@@H](CC(=O)N)C(=O)N)NC(=O)[C@H](Cc1ccccc1)NC(=O)[C@H](CO)[NH3+]
ZINC03830635	-114.16		CC(=O)N(CCCCCNC(=O)CCC(=O)N(CCCCCNC(=O)CCC(=O)N(CCCCC[NH3+])O)O)O
ZINC05411474	-111.71		c1ccc2c(c1)c(c[nH]2)C[C@@H](C(=O)N[C@@H](Cc3c[nH]c4c3cccc4)C(=O)N[C@@H](Cc5c[nH]c6c5cccc6)C(=O)[O-])[NH3+]
ZINC29327528	-110.28		c1cc(cc(c1)C(F)(F)F)CC[C@@H](CC[C@H]2[C@@H](C[C@@H]([C@@H]2CCCCCCC(=O)[O-])O)O)O
ZINC14953257	-110.11		CCCCCCCC/C=C/CCCCCCCC(=O)OC[C@H](COP(=O)([O-])[O-])O
ZINC27215004	-109.83	Compound 4	C[C@H]([C@@H](C(=O)[O-])NC(=O)[C@H](CC(C)C)NC(=O)[C@H](Cc1ccccc1)NC(=O)CNC(=O)[C@@H](CO)NC(=O)[C@H](Cc2ccc(cc2)O)N)O
ZINC08860530	-108.75		CCCCCCCC/C=C\CCCCCCCC(=O)OC[C@H](COP(=O)([O-])[O-])O
ZINC02561119	-108.67	Trp-Gly-Tyr, Compound 8	c1ccc2c(c1)c(c[nH]2)C[C@@H](C(=O)NCC(=O)N[C@@H](Cc3ccc(cc3)O)C(=O)[O-])[NH3+]
ZINC03981882	-107.47		Cc1c(nc(o1)c2ccccc2)COc3ccc(cc3)CO/N=C(\CCC(=O)[O-])/c4ccccc4
ZINC08195621	-105.12		CCCCCCCCCCCCCCCCCCOC[C@H](CO[P@@](=O)([O-])OCC[N+](C)(C)C)OC
ZINC03794794	-104.71		c1cc(c2c(c1NCC[NH2+]CCO)C(=O)c3c(ccc(c3C2=O)O)O)NCC[NH2+]CCO
ZINC14951634	-104.12		c1ccc(cc1)C[C@@H](C(=O)N)NC(=O)[C@H](Cc2ccccc2)NC(=O)[C@@H]3CCCN3C(=O)[C@H](Cc4ccc(cc4)O)N
ZINC03918045	-103.96		CCNC(=O)[C@@H]1[C@H]([C@H]([C@@H](O1)n2cnc3c2nc(nc3N)NCCc4ccc(cc4)CCC(=O)[O-])O)O
ZINC03780900	-103.83		COc1ccc(cc1)/C=C/CCCCOc2ccc(cc2CCC(=O)[O-])C(=O)c3cccc(c3)C(=O)[O-]
ZINC04629873	-103.78		CCCCC/C=C\C/C=C\C=C\C=C\[C@H]([C@H](CCCC(=O)[O-])O)SC[C@@H](C(=O)[O-])[NH3+]
ZINC29134439	-103.34		Cc1cc2c([nH]1)ccc(c2F)Oc3c4c(c(cn4ncn3)OC[C@@H](C)OC(=O)[C@@H](C)[NH3+])C

ZINC04654927	-103.26		CCCC(=O)N[C@@H](Cc1ccc(cc1)O)C(=O)NCCC[NH2+]CCCC[NH2+]CCC[NH3+]
ZINC33359231	-102.03		c1ccc2c(c1)[nH]c(n2)CN(CCCC[NH3+])[C@@H]3CCCc4c3nccc4
ZINC05513160	-101.83		CCCCC[C@@H](/C=C/[C@@H]1[C@H]([C@H](CC1=O)O)C/C=C/CCCC(=O)[O-])O
ZINC14953268	-101.67		CCCCCCCC/C=C/CCCCCCCC(=O)OC[C@@H](COP(=O)([O-])[O-])O
ZINC01893945	-101.67		c1ccc2c(c1)c(c3c([nH+]2)CCCC3)NCCCCCCCNc4c5ccccc5[nH+]c6c4CCCC6
ZINC00841354	-101.49		c1cc(oc1)c2nc3c4cnn(c4nc(n3n2)N)CCCc5ccc(cc5)O
ZINC01530775	-101.06		c1cc(ccc1C(=[NH2+])N)OCCCCCOc2ccc(cc2)C(=[NH2+])N
ZINC26387435	-100.99		CCOC(=O)CNC(=O)[C@H](CSC(=O)N(c1ccc(cc1)Br)O)NC(=O)CC[C@@H](C(=O)OCC)N
ZINC40880490	-100.60		CC(C)(C)OC(=O)Nc1ccc(cc1)c2cc(no2)C(=O)NCCCCCCC(=O)NO
ZINC27076581	-100.60	Compound 5	CC(C)C[C@@H](C(=O)N[C@@H](CC(C)C)C(=O)N[C@@H](CCCNC(=[NH2+])N)C(=O)N)NC(=O)[C@H](Cc1ccccc1)NC(=O)[C@H](CO)[NH3+]
ZINC03930015	-100.46		CC(C)c1c2ccccc2n(c1S(=O)(=O)c3ccc(cc3)OCCC[NH+](C)CCc4ccc(c(c4)OC)OC)C
ZINC03810809	-100.19		CC[NH2+]CCCC[NH2+]CCCC[NH2+]CCCC[NH2+]CCCC[NH2+]CC

Table S2: Virtual screening results using strategy 2.

ZINC ID	VS score	Note	SMILES
ZINC27215004	-142.34	Compound 4	C[C@H]([C@@H](C(=O)[O-])NC(=O)[C@H](CC(C)C)NC(=O)[C@H](Cc1ccccc1)NC(=O)CNC(=O)[C@@H](CO)NC(=O)[C@H](Cc2ccc(cc2)O)N)O
ZINC27076581	-135.69	Compound 5	CC(C)C[C@@H](C(=O)N[C@@H](CC(C)C)C(=O)N[C@@H](CCCNC(=[NH2+])N)C(=O)N)NC(=O)[C@H](Cc1ccccc1)NC(=O)[C@H](CO)[NH3+]
ZINC14951658	-135.13		c1ccc(cc1)C[C@@H](C(=O)N)NC(=O)[C@H](Cc2c[nH]c3c2cccc3)NC(=O)[C@@H]4CCCN4C(=O)[C@H](Cc5ccc(cc5)O)N
ZINC05411474	-133.84		c1ccc2c(c1)c(c[nH]2)C[C@@H](C(=O)N[C@@H](Cc3c[nH]c4c3cccc4)C(=O)N[C@@H](Cc5c[nH]c6c5cccc6)C(=O)[O-])[NH3+]
ZINC37866918	-131.81		Cc1cc(ccc1OCC(=O)[O-])OCc2cc(cc(c2)c3ccc(cc3)C(F)(F)F)c4ccc(cc4)C(F)(F)F
ZINC45315721	-128.28		CCOC(=O)CCc1ccc(c(c1)C#N)OC[C@@H](C[NH2+]C(C)(C)Cc2ccc3ccccc3c2)O
ZINC28571624	-128.03		Cc1ccc(cc1Nc2nccc(n2)c3cncnc3)NC(=O)c4ccc(c(c4)C(F)(F)F)C[NH+]5CC[C@H](C5)[NH+](C)C
ZINC14276972	-127.75		CCCCCCCCCCOc1cc(cc(c1)OCCCCCCCCCC)N(CC(=O)[O-])CC(=O)[O-]
ZINC29327528	-127.47		c1cc(cc(c1)C(F)(F)F)CC[C@@H](CC[C@H]2[C@@H](C[C@@H]([C@@H]2CCCCCCC(=O)[O-])O)O)O
ZINC03830635	-126.93		CC(=O)N(CCCCCNC(=O)CCC(=O)N(CCCCCNC(=O)CCC(=O)N(CCCCC[NH3+])O)O)O
ZINC14951634	-126.24		c1ccc(cc1)C[C@@H](C(=O)N)NC(=O)[C@H](Cc2ccccc2)NC(=O)[C@@H]3CCCN3C(=O)[C@H](Cc4ccc(cc4)O)N
ZINC01550477	-124.87		CS(=O)(=O)CC[NH2+]Cc1ccc(o1)c2ccc3c(c2)c(ncn3)Nc4ccc(c(c4)Cl)OCc5cccc(c5)F
ZINC14972488	-124.38		c1ccc(cc1)Cc2cnc3c(c2c4cccc(c4)OCc5ccc(cc5)CC(=O)[O-])cccc3C(F)(F)F

ZINC0 46298 73	- 124 .31		CCCCC/C=C\C/C=C\C=C\C=C\[C@H]([C@H](CCCC(=O)[O-])O)SC[C@@H](C(=O)[O-])[NH3+]
ZINC0 38113 14	- 123 .10		c1ccc(cc1)C(c2ccccc2)C(=O)N[C@H](CCCNC(=[NH2+])N)C(=O)NCc3ccc(cc3)O
ZINC2 66419 17	- 122 .24		COc1ccc2c(c1)c(c3ccc(cc3[nH+]2)Cl)NCCC[NH+]4CCN(CC4)CCCNc5c6ccc(cc6[nH+]c7c5cc(cc7)OC)Cl
ZINC0 39525 32	- 121 .83		CC(C)(C/C=C/C(=O)N(C)[C@H](Cc1ccc2ccccc2c1)C(=O)N(C)[C@H](Cc3ccccc3)C(=O)NC)[NH3+]
ZINC4 28343 88	- 121 .63		CNC(=O)c1cccc(c1)c2ccc(cc2)[C@H](CN3CCOCC3)N(C)C(=O)Cn4c5cc(c(cc5ncc4=O)Cl)Cl
ZINC2 92215 75	- 121 .13		CC(C)C[C@@H](C(=O)N[C@@H](CC(C)C)C(=O)N[C@@H](CCCNC(=[NH2+])N)C(=O)N[C@@H](CC(=O)N)C(=O)N)NC(=O)[C@H](Cc1ccccc1)NC(=O)[C@H](CO)[NH3+]
ZINC0 81956 21	- 121 .10		CCCCCCCCCCCCCCCCCCOC[C@H](CO[P@@](=O)([O-])OCC[N+](C)(C)C)OC
ZINC0 39818 82	- 121 .01		Cc1c(nc(o1)c2ccccc2)COc3ccc(cc3)CO/N=C(\CCC(=O)[O-])/c4ccccc4
ZINC0 97817 93	- 120 .67		C=CCc1ccccc1OC[C@H](C[NH+]2CCC(CC2)CN3C(=O)c4cccc5c4c(ccc5)C3=O)O
ZINC0 37875 91	- 120 .23		CN([C@H](C[NH+]1CCCC1)c2cccc(c2)OCC(=O)[O-])C(=O)Cc3ccc(c(c3)Cl)Cl
ZINC0 37947 94	- 119 .51		c1cc(c2c(c1NCC[NH2+]CCO)C(=O)c3c(ccc(c3C2=O)O)O)NCC[NH2+]CCO
ZINC1 37199 16	- 119 .46		c1ccc(cc1)c2ccc(cc2)CO[C@H]3C[C@@H]([C@@H]([C@H]3CC/C=C\CCC(=O)[O-])[NH+]4CCCCC4)O
ZINC4 28343 86	- 118 .91		CNC(=O)c1cccc(c1)c2ccc(cc2)[C@@H](CN3CCOCC3)N(C)C(=O)Cn4c5cc(c(cc5ncc4=O)Cl)Cl
ZINC0 46548 75	- 118 .74		c1ccc2c(c1)cccc2CC(=O)NCCC[NH2+]CCCC[NH2+]CCC[NH3+]

ZINC000602799	-118.15		Cc1c2cc(ccc2n(c1c3ccc(cc3)O)Cc4ccc(cc4)OCC[NH+]5CCCCC5)O
ZINC003973581	-118.04		CCCC[NH+](CCC1CCCCC1)Cc2ccc(c(c2)c3ccccc3C)C(=O)N[C@@H](CCSC)C(=O)[O-]
ZINC29136330	-118.03		c1ccc(cc1)Oc2ccc(cc2)C(=O)c3cc(c(c(c3[O-])O)[O-])C(=O)c4nc5ccccc5s4
ZINC001530850	-117.50		COc1cc(ccc1O)[C@H]2[C@@H](Oc3ccc(cc3O2)[C@@H]4[C@H](C(=O)c5c(cc(cc5O4)O)O)O)CO
ZINC13741955	-117.42		CCCc1c(ccc(c1O)C(=O)C)OC[C@H](COc2ccc3c(=O)cc(oc3c2CCC)C(=O)[O-])O
ZINC003872327	-117.23		Cn1cc(cc1C(=O)Nc2cc(n(c2)C)C(=O)Nc3cc(n(c3)C)C(=O)NCCC(=[NH2+])N)NC=O
ZINC42919869	-117.07		Cc1ccc(cc1/C=C/n2cnc3c2ncnc3NC4CC4)C(=O)Nc5cc(cc(c5)n6cc([nH+]c6)C)C(F)(F)F
ZINC001914818	-116.84		CC(C)(C)Sc1c2cc(ccc2n(c1CC(C)(C)C(=O)[O-])Cc3ccc(cc3)Cl)OCc4ccc5ccccc5n4
ZINC26835202	-116.83		C#CCN(Cc1ccc2c(c1)c(=O)[nH]c(n2)N)c3ccc(c(c3)F)C(=O)N[C@@H](CCC(=O)[O-])C(=O)[O-]
ZINC005723394	-116.26		CC[NH+](CC)C[C@@H](CNc1ccc(c2c1C(=O)c3ccccc3C2=O)NC[C@H](CCl)O)O
ZINC19217280	-116.21		C#CCN(Cc1ccc2c(c1)c(=O)[nH]c(n2)N)c3ccc(cc3)C(=O)N[C@@H](CCC(=O)[O-])C(=O)[O-]
ZINC25973342	-115.98		CC(C)C[C@@H](C(=O)N[C@@H](Cc1ccccc1)C(=O)[O-])NC(=O)[C@H](CCSC)NC(=O)Nc2ccc(cc2)OC
ZINC38149756	-115.77		c1ccc(cc1)C(=O)Cn2c(=O)ccn(c2=O)[C@H]3[C@@H]([C@@H]([C@H](O3)CO[P@@](=O)([O-])OP(=O)([O-])[O-])O)O
ZINC004172316	-115.73		CCCc1c(ccc(c1O)C(=O)C)OCCCCCOc2cc3c(cc2C(=O)C)CC[C@H](O3)C(=O)[O-]

ZINC01914818	-115.68		CC(C)(C)Sc1c2cc(ccc2n(c1CC(C)(C)C(=O)[O-])Cc3ccc(cc3)Cl)OCc4ccc5ccccc5n4
ZINC49878561	-115.60		CC[C@H](C(=O)[O-])Oc1cccc(c1)n2c(c(c3c2cc(cc3)OC(F)(F)F)C(=O)c4ccc(cc4)OC)C
ZINC08022594	-115.57		Cc1cc(ccc1OCC(=O)[O-])SCc2c(nc(s2)c3ccc(c(c3)F)C(F)(F)F)C
ZINC32785912	-115.52		CCCCCCCCCCCCC/C=C/[C@@H]([C@H](COP(=O)([O-])[O-])[NH3+])O
ZINC03872994	-115.40		CC(=O)N1CCN(CC1)c2ccc(cc2)OC[C@H]3CO[C@@](O3)(Cn4cc[nH+]c4)c5ccc(cc5Cl)Cl
ZINC02007560	-115.36		CC[NH+](CC)CCCCNc1c(nc2cc(c(cc2n1)Cl)Cl)c3ccc(s3)c4cccs4
ZINC01886617	-115.15		CC(C)n1c2ccccc2c(c1/C=C/[C@H](C[C@H](CC(=O)[O-])O)O)c3ccc(cc3)F
ZINC11676975	-115.14		CCCc1c(ccc(c1O)C(=O)C)OCCCCCOc2cc3c(cc2C(=O)C)CC[C@@H](O3)C(=O)[O-]
ZINC08860500	-115.06		CCCCCCCCCCCCC/C=C/[C@H]([C@H](COP(=O)([O-])[O-])[NH3+])O
ZINC27519870	-114.83		CN(C)c1ccc(cc1)Nc2c3ccc(cc3[nH+]c4c2ccc(c4)NC(=O)CC[NH+]5CCCC5)NC(=O)CC[NH+]6CCCC6
ZINC01613467	-114.77		C[NH+](C)CCNC(=O)c1cccc2c1nc(cc2)c3ccc(cc3)c4ccccc4
ZINC72117104	-114.67		CC[C@H](C)[C@@H](C(=O)N1Cc2cc(ccc2C[C@H]1C(=O)Nc3ccc(cc3)OC)OCC(=O)NO)NC(=O)CC(C)(C)C
ZINC04654927	-114.40		CCCC(=O)N[C@@H](Cc1ccc(cc1)O)C(=O)NCCC[NH2+]CCCC[NH2+]CCC[NH3+]
ZINC14953257	-114.00		CCCCCCCC/C=C/CCCCCCCC(=O)OC[C@H](COP(=O)([O-])[O-])O

ZINC03927570	-113.84		C(C[C@@H](C(=O)NCC(=O)N[C@@H](CC(=O)[O-])C(=O)N[C@@H](CO)C(=O)[O-])[NH3+])CNC(=[NH2+])N
ZINC20148990	-113.74		Cc1ccc(cc1c2ccc3c(c2)cnc(n3)NCCN4CCOCC4)C(=O)NC5CC5
ZINC13741956	-113.40		CCCc1c(ccc(c1O)C(=O)C)OC[C@@H](COc2ccc3c(=O)cc(oc3c2CCC)C(=O)[O-])O
ZINC24530552	-113.36		CC(=O)N[C@@H]1[C@H]([C@H]([C@H](O[C@@H]1O[P@@](=O)([O-])O[P@@](=O)([O-])OC[C@@H]2[C@H]([C@H]([C@@H](O2)n3ccc(=O)[nH]c3=O)O)O)CO)O)O
ZINC00643046	-113.24		C1CCN(CC1)c2c3c(c(nc(n3)N(CCO)CCO)N4CCCCC4)nc(n2)N(CCO)CCO
ZINC03807186	-113.18		c1ccc(c(c1)C(=O)NCCC[C@@H](C(=O)[O-])NC(=O)c2ccc(cc2)NCc3cnc4c(n3)c(nc(n4)N)N)C(=O)[O-]
ZINC03798763	-113.16		CC[C@H](C)C(=O)O[C@H]1C[C@@H](C=C2[C@H]1[C@H]([C@H](C=C2)C)CC[C@H](C[C@H](CC(=O)[O-])O)O)O
ZINC03871704	-113.08		CC#CC[C@@H](C)[C@@H](/C=C/[C@@H]1[C@@H](C[C@@H]2[C@@H]1c3cccc(c3O2)CCCC(=O)[O-])O)O
ZINC30320690	-112.90		c1cn(c(=O)[nH]c1=O)[C@H]2[C@@H]([C@@H]([C@H](O2)CO[P@@](=O)([O-])O[P@@](=O)([O-])O[C@@H]3[C@@H]([C@H]([C@@H]([C@H](O3)C(=O)[O-])O)O)O)O)O
ZINC02541693	-112.85		CCCc1c(ccc(c1O)C(=O)C)OCCCOc2ccc(cc2)OCC(=O)[O-]
ZINC30320665	-112.83		c1cn(c(=O)[nH]c1=O)[C@H]2[C@@H]([C@@H]([C@H](O2)CO[P@](=O)([O-])O[P@](=O)([O-])O[C@@H]3[C@@H]([C@H]([C@@H]([C@H](O3)CO)O)O)O)O)O
ZINC04654969	-112.83		CCCCC/C=C\C/C=C\C/C=C\C/C=C\CCCC(=O)OC(CO)CO
ZINC03871706	-112.83		CC#CC[C@H](C)[C@@H](/C=C/[C@@H]1[C@@H](C[C@@H]2[C@@H]1c3cccc(c3O2)CCCC(=O)[O-])O)O
ZINC03996003	-112.82		C[NH+](CC[C@H](c1ccc(cc1)F)Oc2ccc(cc2)c3ccccc3)CC(=O)[O-]

ZINC0 16257 51	- 112 .79		Cc1[nH]c(=O)c2cc(ccc2n1)CN(C)c3ccc(s3)C(=O)N[C@H](CCC(=O)[O-])C(=O)[O-]
ZINC1 32843 73	- 112 .74		CN(Cc1cnc2c(n1)c(nc(n2)N)N)c3ccc(cc3)C(=O)N[C@@H](CCCC[NH3+])C(=O)[O-]
ZINC0 38828 97	- 112 .27		COc1ccc2c(n1)c(c3ccc(cc3n2)Cl)Nc4cc(c(c(c4)C[NH+]5CCCC5)O)C[NH+]6CCCC6
ZINC0 39194 14	- 112 .13		CC(C)[C@@H](C(=O)[O-])NC(=O)[C@H](CC(=O)[O-])NC(=O)CNC(=O)[C@H](CCCNC(=[NH2+])N)[NH3+]
ZINC1 49532 68	- 112 .12		CCCCCCCC/C=C/CCCCCCCC(=O)OC[C@@H](COP(=O)([O-])[O-])O
ZINC0 25611 19	- 112 .01	Trp-Gly-Tyr, Compound 6	c1ccc2c(c1)c(c[nH]2)C[C@@H](C(=O)NCC(=O)N[C@@H](Cc3ccc(cc3)O)C(=O)[O-])[NH3+]
ZINC0 15499 89	- 111 .74		Cc1cc(ccc1OCC(=O)[O-])SCc2c(nc(s2)c3ccc(cc3)C(F)(F)F)C
ZINC0 39527 25	- 111 .64		C[C@H](Cc1c[nH]c2c1cccc2OCC(=O)[O-])[NH2+]C[C@@H](c3cccc(c3)Cl)O
ZINC2 68446 84	- 111 .19		CCC#CC[C@H](C)[C@@H](C#C[C@H]1[C@@H](C[C@@H]2[C@@H]1C/C(=C/COCC(=O)[O-])/C2)O)O
ZINC0 40962 58	- 111 .15		c1cc(c(cc1c2cc(=O)c3c(cc(cc3o2)O[C@H]4[C@@H]([C@H]([C@@H]([C@H](O4)CO)O)O)O)O)O)O
ZINC0 20335 89	- 110 .71		COc1cc(ccc1O)[C@@H]2[C@H](Oc3ccc(cc3O2)[C@@H]4[C@H](C(=O)c5c(cc(cc5O4)O)O)O)CO
ZINC3 33592 31	- 109 .33		c1ccc2c(c1)[nH]c(n2)CN(CCCC[NH3+])[C@@H]3CCCc4c3nccc4
ZINC0 38091 92	- 107 .84		CC(C)CN(C[C@H]([C@H](Cc1ccccc1)NC(=O)O[C@H]2CCOC2)O)S(=O)(=O)c3ccc(cc3)N
ZINC0 38308 63	- 106 .85		CC(C)n1c2ccccc2c(c1/C=C/[C@H](C[C@@H](CC(=O)[O-])O)O)c3ccc(cc3)F

ZINC0 38308 64	- 106 .01		CC(C)n1c2ccccc2c(c1/C=C/[C@@H](C[C@H](CC(=O)[O-])O)O)c3ccc(cc3)F
ZINC0 06079 71	- 105 .66		c1cc(c(cc1Cl)Cl)CO[C@H](Cn2cc[nH+]c2)c3ccc(cc3Cl)Cl
ZINC0 42122 06	- 105 .62		CC(C)(Cc1c[nH]c2c1cccc2)[NH2+]C[C@@H](COc3ccccc3C#N)O
ZINC0 15307 75	- 105 .03		c1cc(ccc1C(=[NH2+])N)OCCCCCOc2ccc(cc2)C(=[NH2+])N
ZINC0 08967 40	- 104 .01		c1cc(c(cc1Cl)Cl)CO[C@@H](Cn2cc[nH+]c2)c3ccc(cc3Cl)Cl
ZINC0 05377 95	- 103 .86		Cc1cnc(cn1)C(=O)NCCc2ccc(cc2)S(=O)(=O)/N=C(/NC3CCCCC3)\[O-]
ZINC0 06430 55	- 103 .28		c1cc(ccc1CO[C@@H](Cn2cc[nH+]c2)c3ccc(cc3Cl)Cl)Cl
ZINC0 15571 61	- 102 .71		c1ccc2c(c1)c(c[nH]2)C[C@@H](C(=O)N[C@@H](Cc3c[nH]c4c3cccc4)C(=O)[O-])[NH3+]
ZINC0 16656 51	- 102 .54		c1cc(c(cc1C(=[NH2+])N)Br)OCCCOc2ccc(cc2Br)C(=[NH2+])N
ZINC0 15293 23	- 102 .19		CN(Cc1cnc2c(n1)c(nc(n2)N)N)c3ccc(cc3)C(=O)N[C@@H](CCC(=O)[O-])C(=O)[O-]
ZINC0 16655 64	- 101 .98		c1cc(ccc1C(=[NH2+])N)OCCCOc2ccc(cc2)C(=[NH2+])N
ZINC0 05968 81	- 101 .45		c1cc(ccc1CO[C@H](Cn2cc[nH+]c2)c3ccc(cc3Cl)Cl)Cl
ZINC0 48994 65	- 100 .12		CSCC[C@@H](C(=O)N[C@@H](CCSC)C(=O)N[C@@H](CCSC)C(=O)[O-])[NH3+]

Table S3: List of synthesized peptides from VS

PEPTIDE	SEQUENCE	MW	YIELD (%)	T_R (MIN)	K_D (µM)
**1**	wWW	576.64	31	16.94	No binding
**2**	wGGY	481.50	28	11.06	No binding
**3**	YPFF-$CONH_2$	571.67	29	13.93	No binding
**4**	YSGFLT	686.75	33	16.00	18±1
**5**	SFLLR-$CONH_2$	633.80	39	11.98	44±3
**6**	wGy	424.45	48	15.38	3±1
**7**	SFLLRN-$CONH_2$	747.89	21	14.98	No binding
**8**	WGY	424.45	23	15.51	No binding
**9**	YPWF-$CONH_2$	610.70	26	14.36	No binding

__Microscale thermophoresis results for binding peptides.__

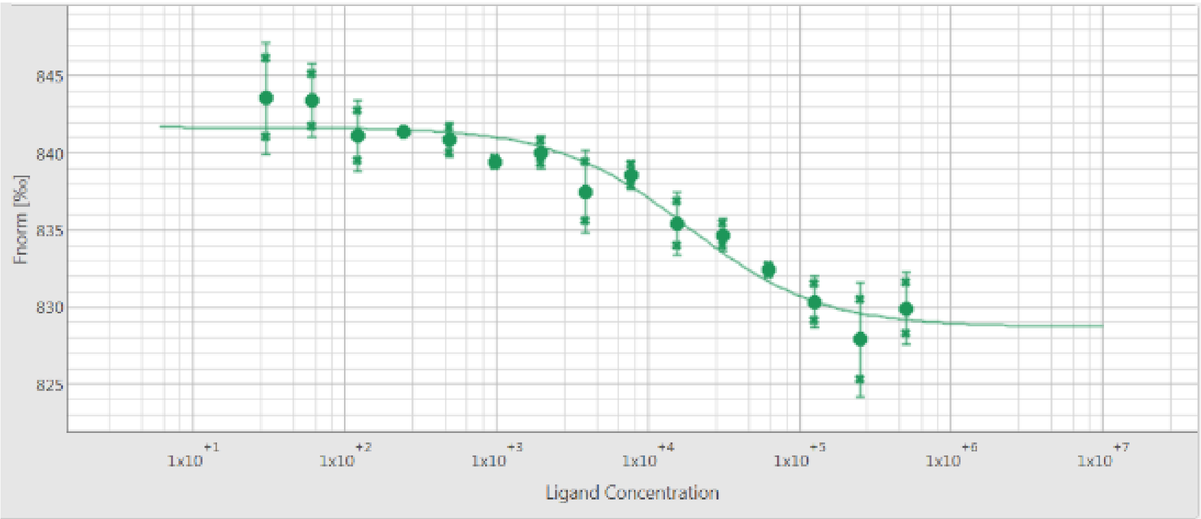

PARAMETER	RESULT
KD	18.299 µM
STANDARD ERROR	1.2809

Figure S2: Binding affinity profile for peptide **4**.

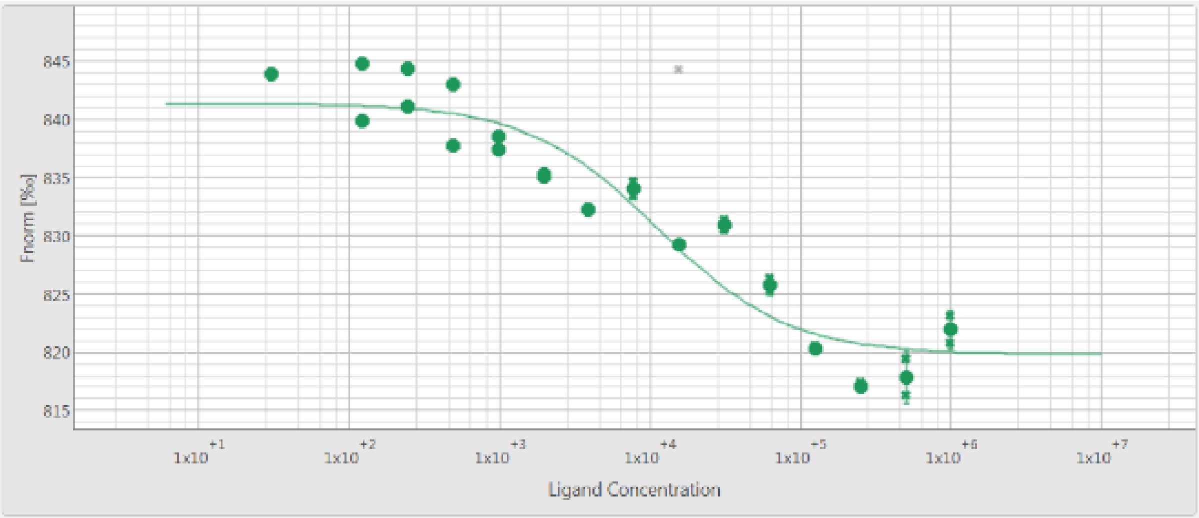

PARAMETER	RESULT
KD	44.366 µM
STANDARD ERROR	2.6667

Figure S3: Binding affinity profile for peptide **5.**

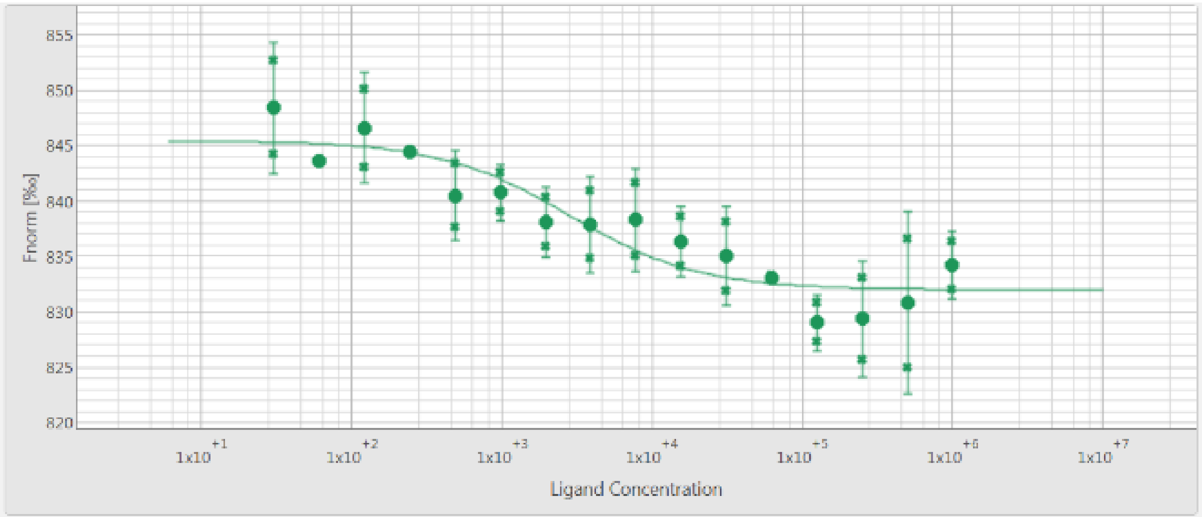

PARAMETER	RESULT
**KD**	2.7892 µM
**STANDARD ERROR**	1.4594

Figure S4: Binding affinity profile for peptide **6**.

Table S4: Summary of the Hill equation parameters for PFKFB3 alone or in presence of the tested compounds

compound	V_{max}	±	S.E.	K_{half}	±	S.E.	h	±	S.E.
**none**	0.16	±	0.03	21.51	±	5.57	2.03	±	0.66
**4**	0.18	±	0.04	26.54	±	5.71	2.39	±	0.68
**5**	0.81	±	0.29	58.42	±	25.88	1.50	±	0.21
**6**	0.91	±	0.43	71.72	±	44.97	1.30	±	0.20
**7**	0.19	±	0.06	28.12	±	10.95	1.95	±	0.69
**8**	0.21	±	0.09	32.83	±	15.32	2.24	±	0.96
**AZ33 (1)**	1.06	±	1.41	94.02	±	169.25	1.20	±	0.40

Characterization of compounds 1-9.

1 (wWW)**:** white powder; yield: 31%; calculated MW: 576.64, found MS (ESI) *m/z* = 575.72 ([M-H]$^-$); HPLC: tr= 16.94 min, 100 % at 280 nm, Method A (gradient elution of 5–70% solvent B (solvent A: water/acetonitrile/TFA95/5/0.1; solvent B: water/acetonitrile/TFA 5/95/0.1) over 20 min at a flowrate of 0.8 ml/min)).

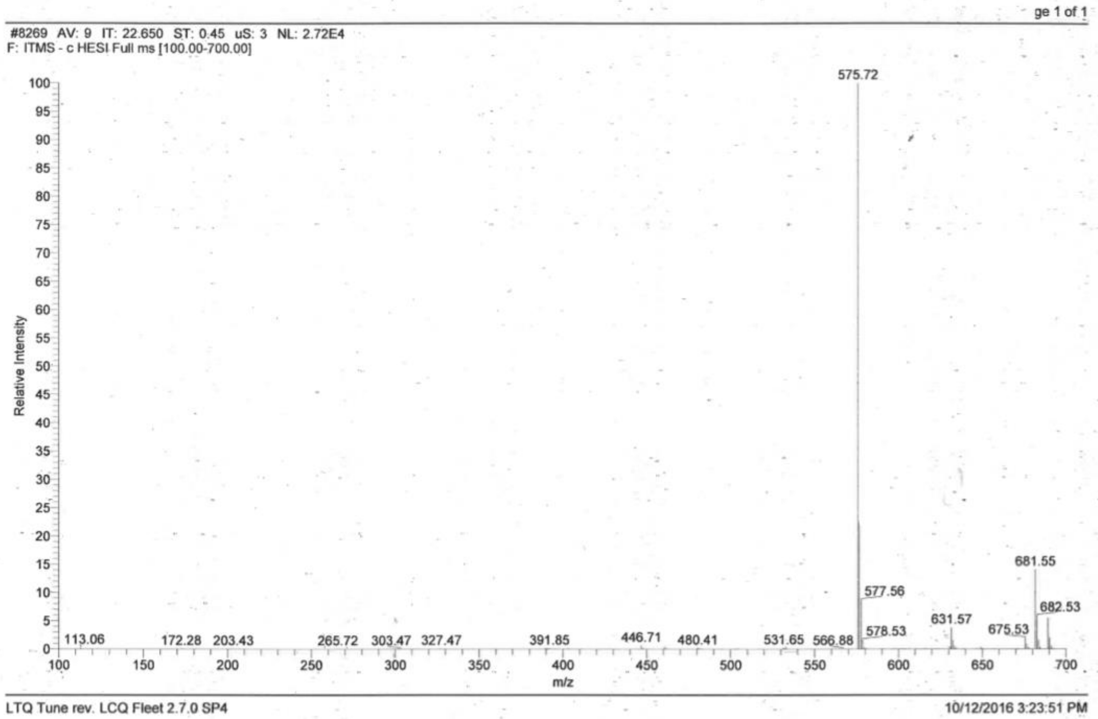

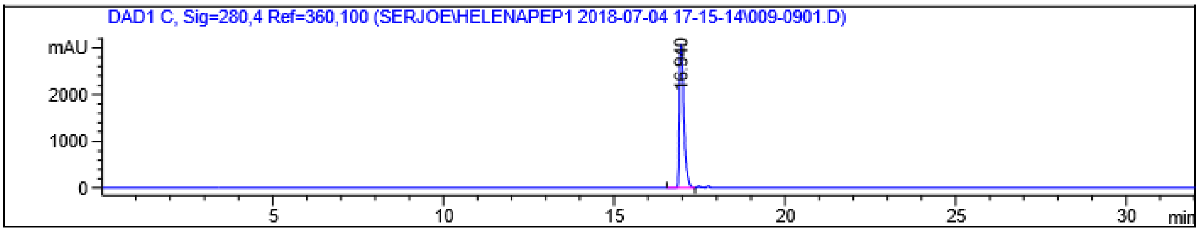

2 (wGGY)**:** white powder; yield: 25%; calculated MW: 481.50, found: MS (ESI) *m/z* = 480.6 ([M-H]$^-$); HPLC: tr= 11.056 min, 96.5 % at 280 nm, Method A.

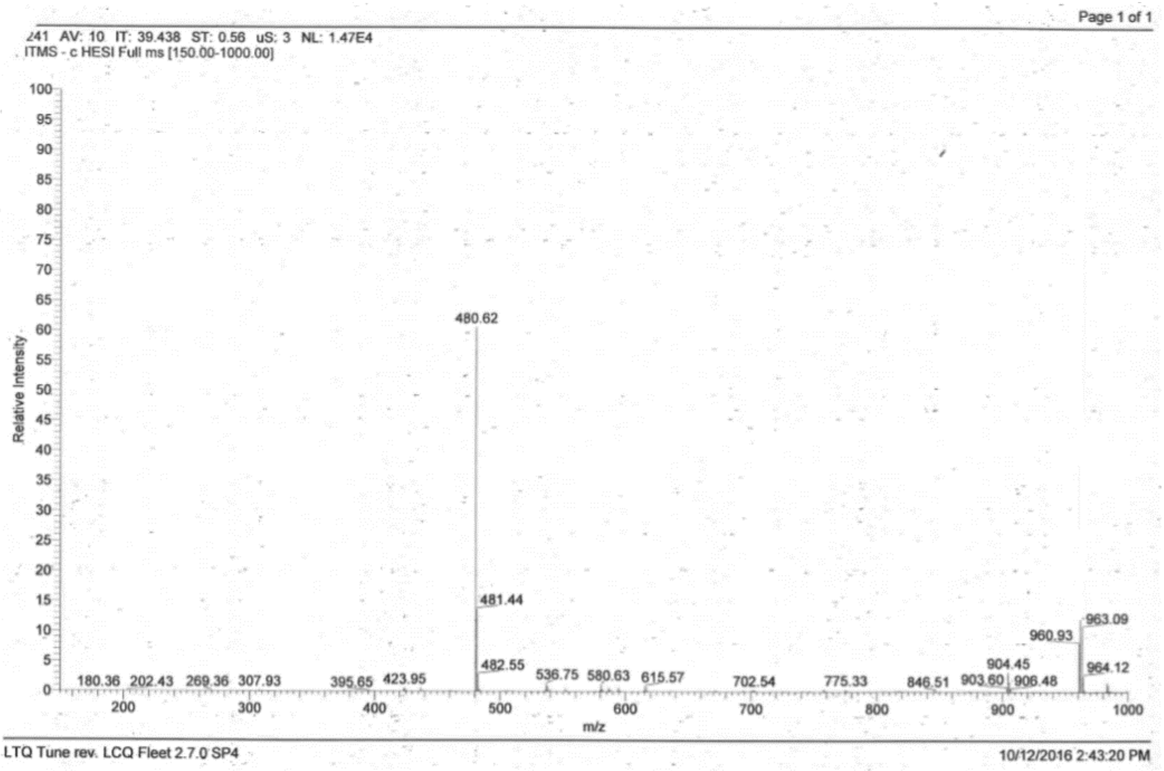

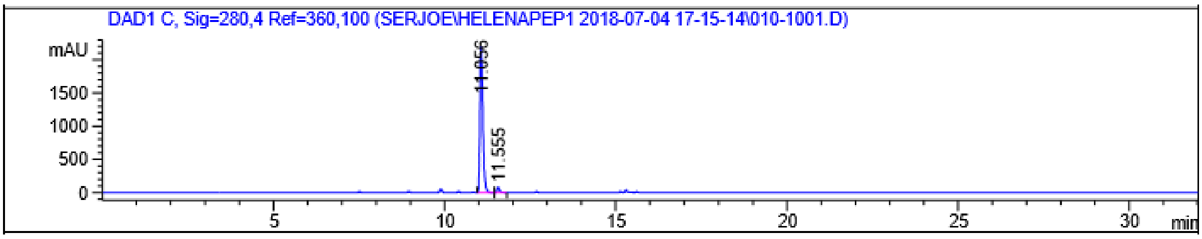

3 (YPFF-CONH2)**:** white powder; yield: 29%; calculated MW: 571.67, found: MS (ESI) *m/z* = 572.5 ([M+H]$^+$); HPLC: tr=13.93 min, 100 % at 220 nm, Method A.

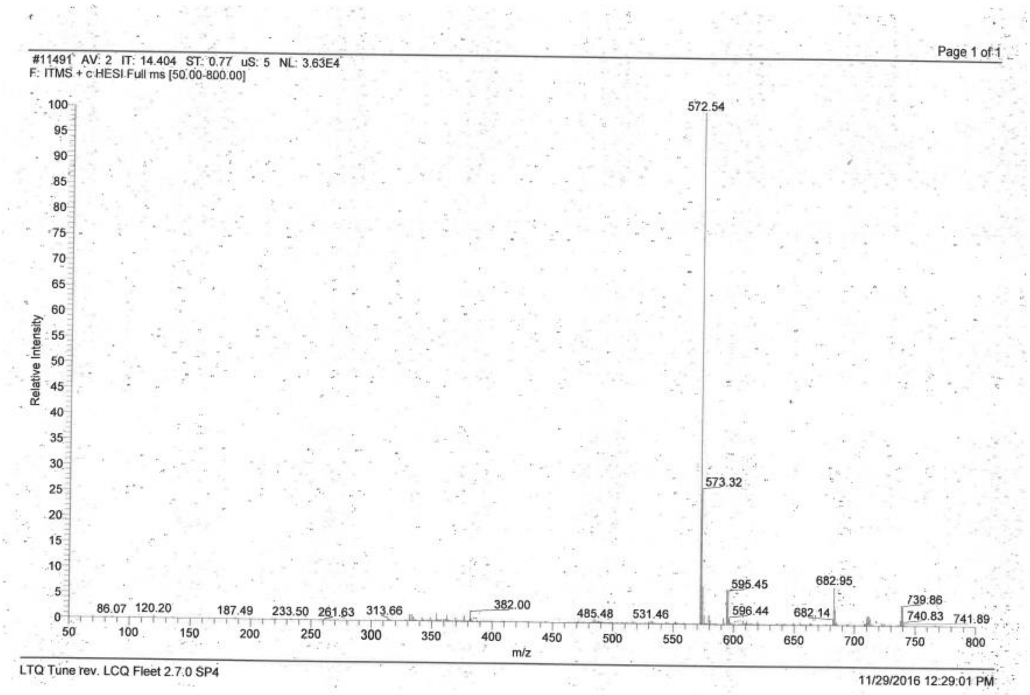

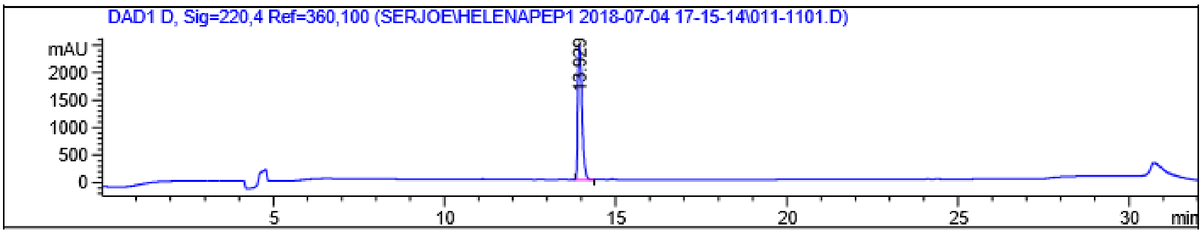

4 (YSGFLT)**:** white powder; yield: 33%; calculated MW: 686.75, found: MS (ESI) m/z = 687.5 ($[M+H]^+$), HRMS ($[M+H]^+$) for $C_{33}H_{46}N_6O_{10}$: calculated 687.3356, found 687.3354; HPLC: t_r = 16.00 min; 96.9% at 220 nm, Method A.

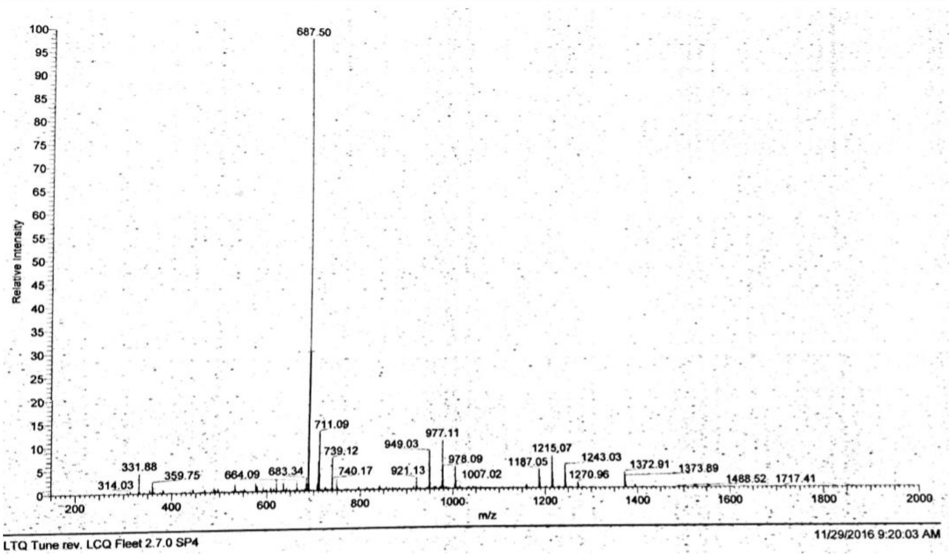

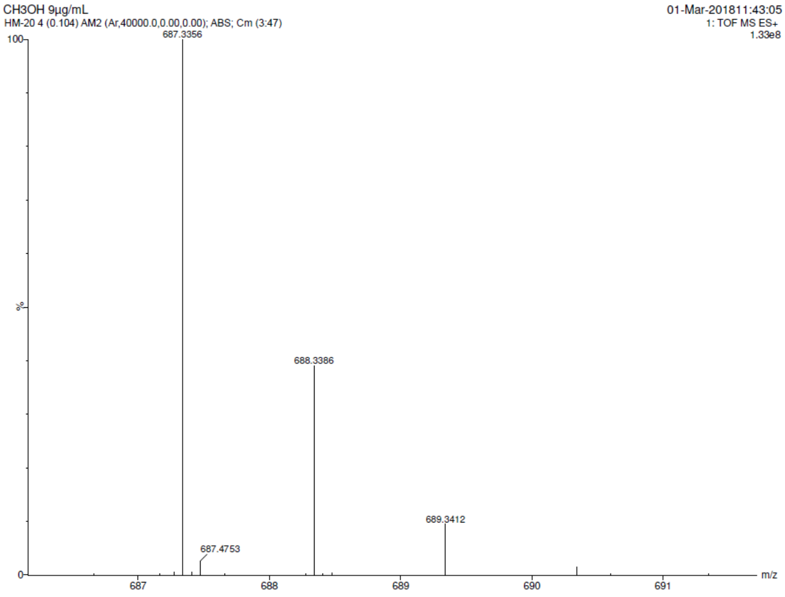

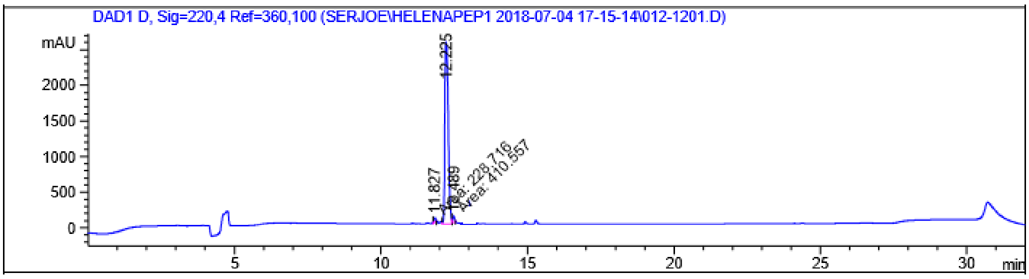

5 (SFLLR-CONH$_2$)**:** white powder; yield: 39%; calculated MW: 633.78, found: MS (ESI) *m/z* = 634.7 ([M+H]$^+$), HRMS ([M+H]$^+$) for $C_{30}H_{53}N_9O_6$: calculated 634.4037, found 634.4041; HPLC: tr= 11.98 min; 98.41 % at 220 nm, Method A.

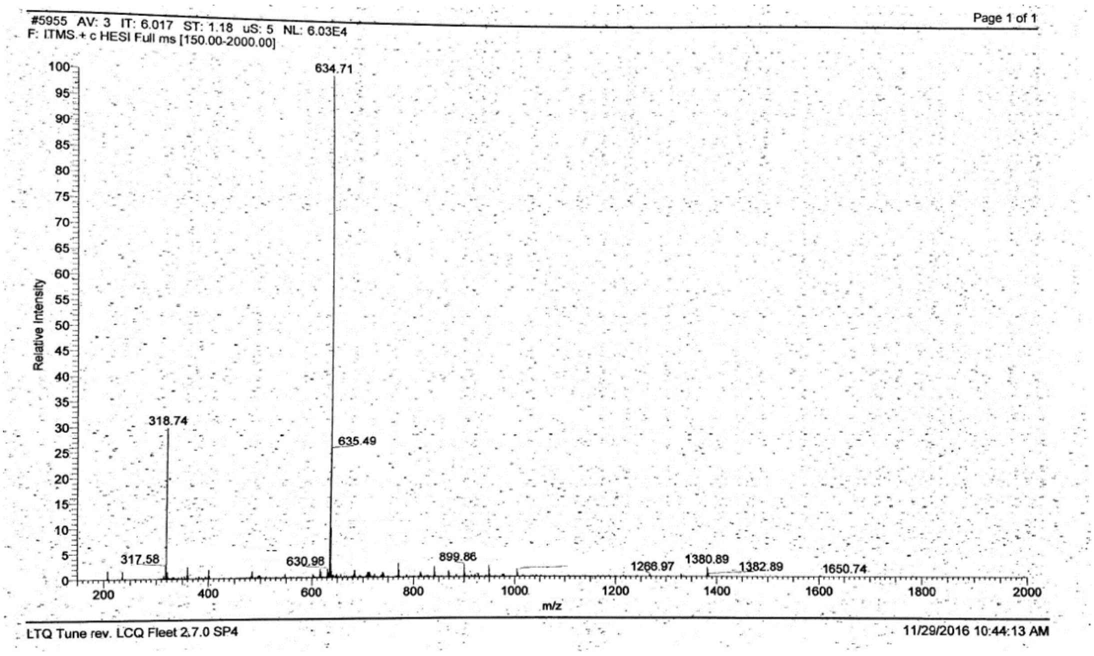

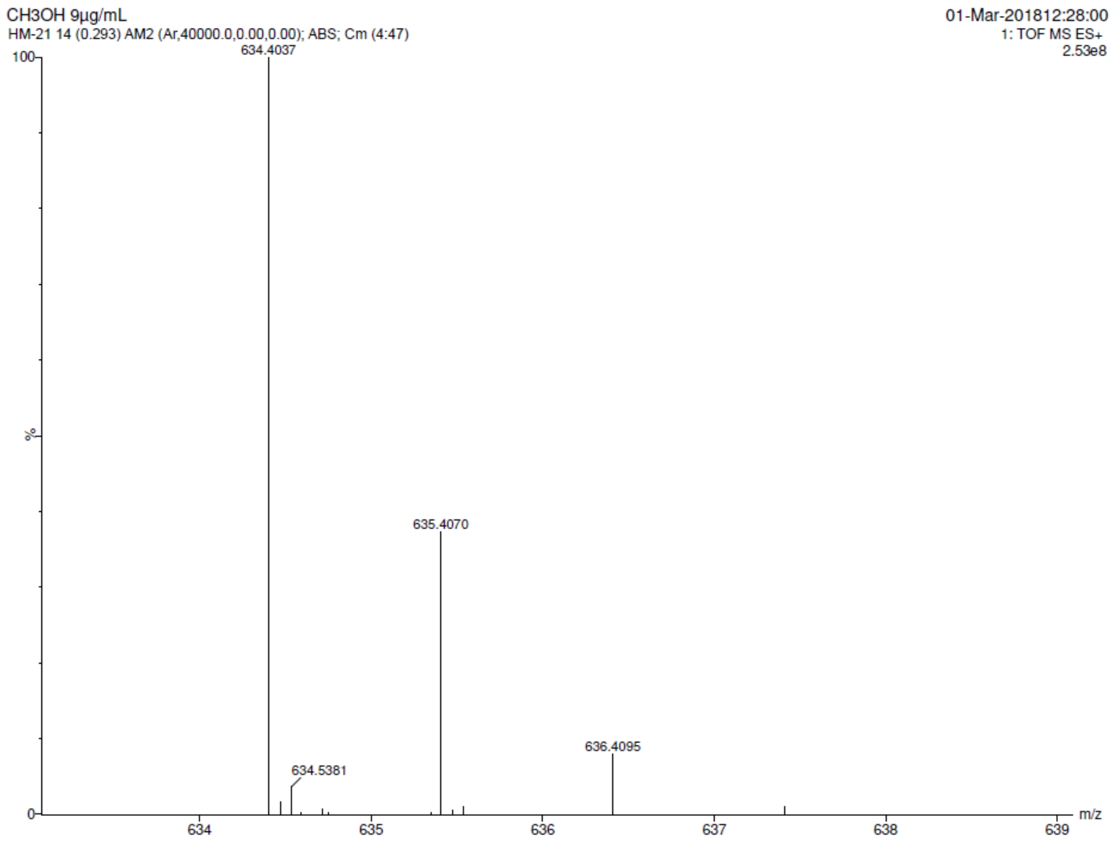

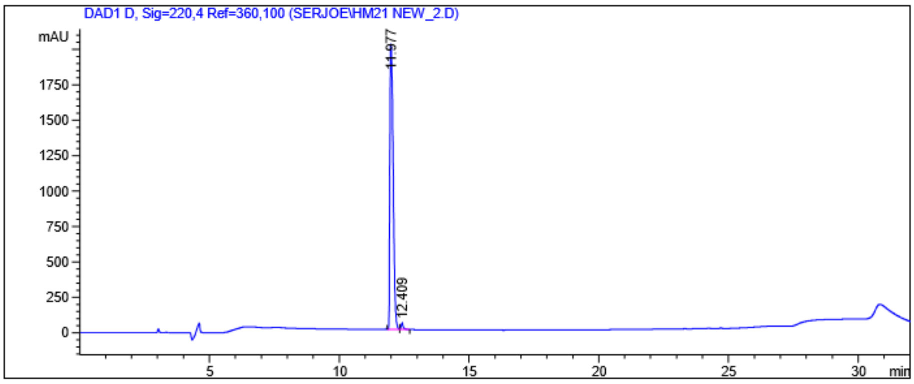

6 (wGy)**:** white powder; yield: 48%; calculated MW: 424.45, found: MS (ESI) *m/z* = 425.68 ([M+H]$^+$), HRMS ([M-H]$^-$) for $C_{22}H_{24}N_4O_5$: calculated 423.1667, found 423.1668; HPLC: t_r = 15.38 min, 100 % at 220 nm, Method A.

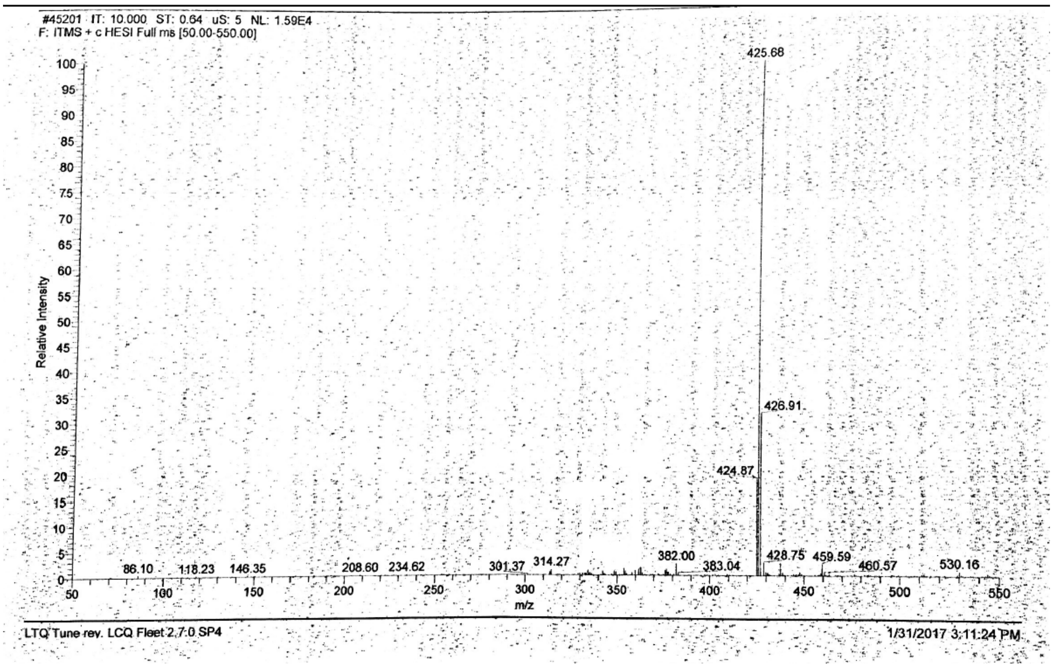

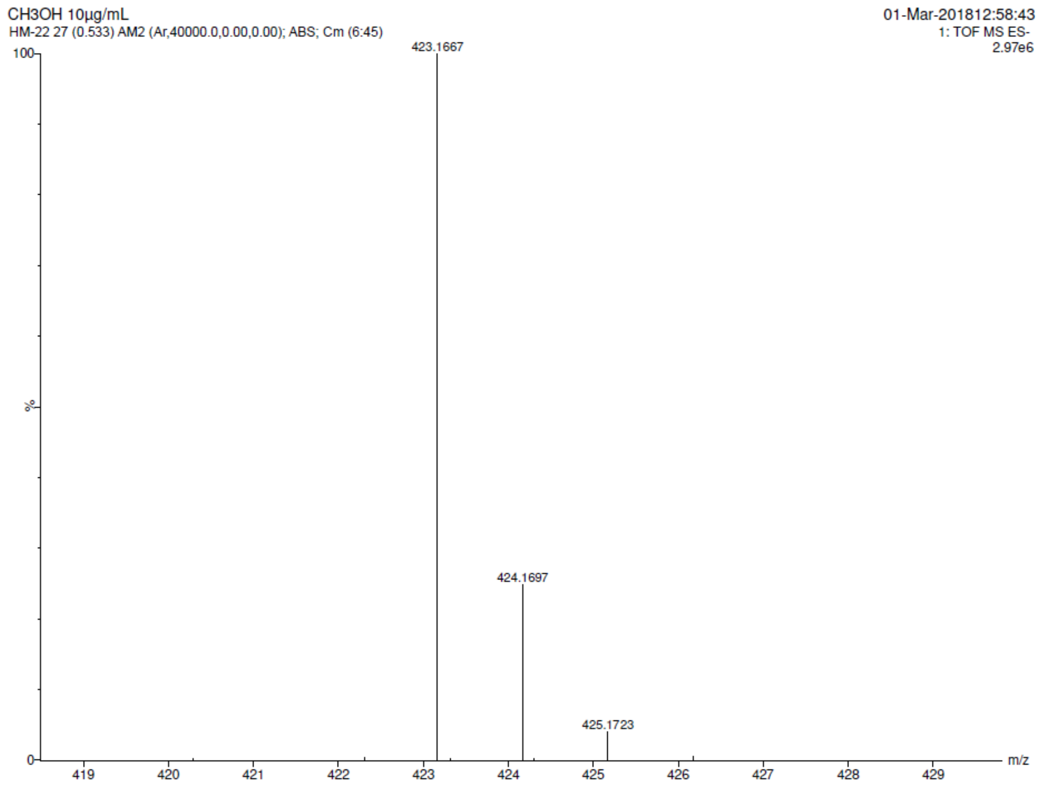
CH3OH 10µg/mL
HM-22 27 (0.533) AM2 (Ar,40000.0,0.00,0.00); ABS; Cm (6:45)
01-Mar-201812:58:43
1: TOF MS ES-
2.97e6
423.1667
424.1697
425.1723
100
%
0
419
420
421
422
423
424
425
426
427
428
429
m/z

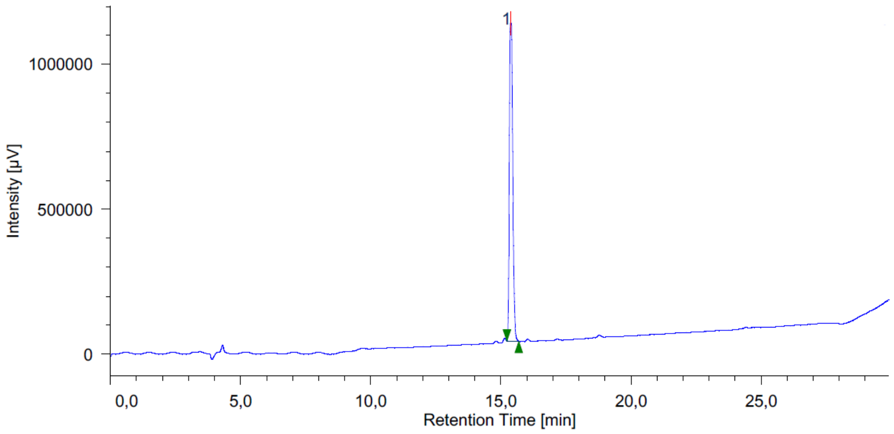
1
1000000
500000
0
Intensity [µV]
0,0
5,0
10,0
15,0
20,0
25,0
Retention Time [min]

7 (SFLLRN-CONH2)**:** white powder; yield: 21%; calculated MW: 747.89, found: MS (ESI) *m/z* = 748.9 ($[M+H]^+$), HRMS (($[M+H]^+$) for $C_{34}H_{59}N_{11}O_8$: calculated 748.4469, found 748.4467; HPLC: t_r = 14.98 min, 100 % at 220 nm, Method A.

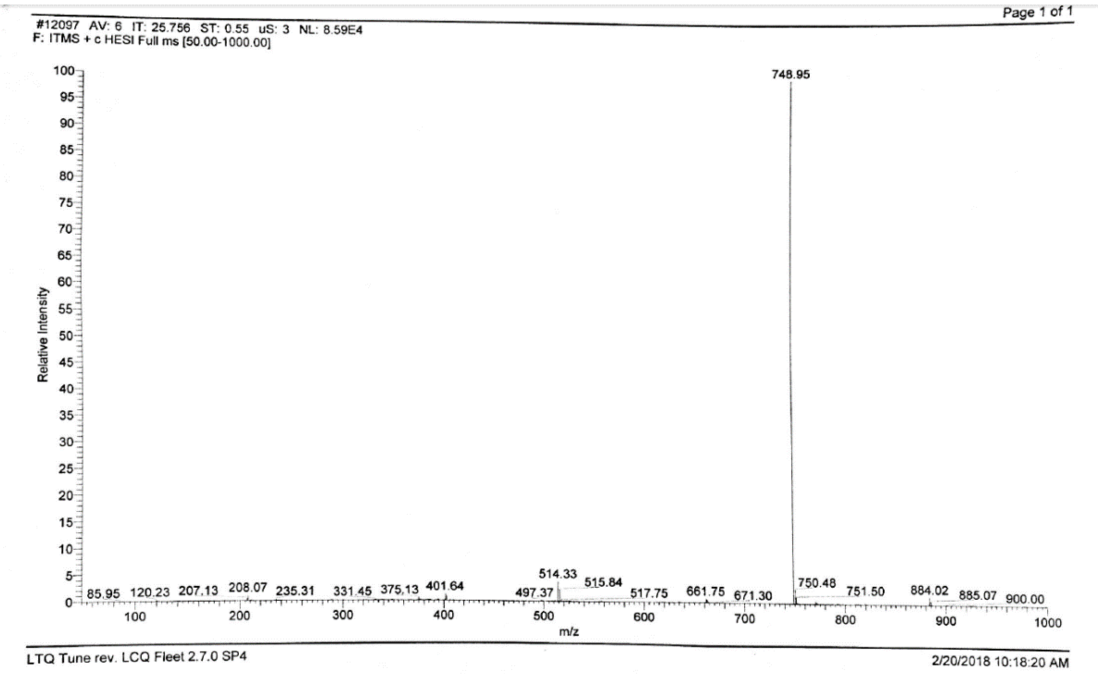

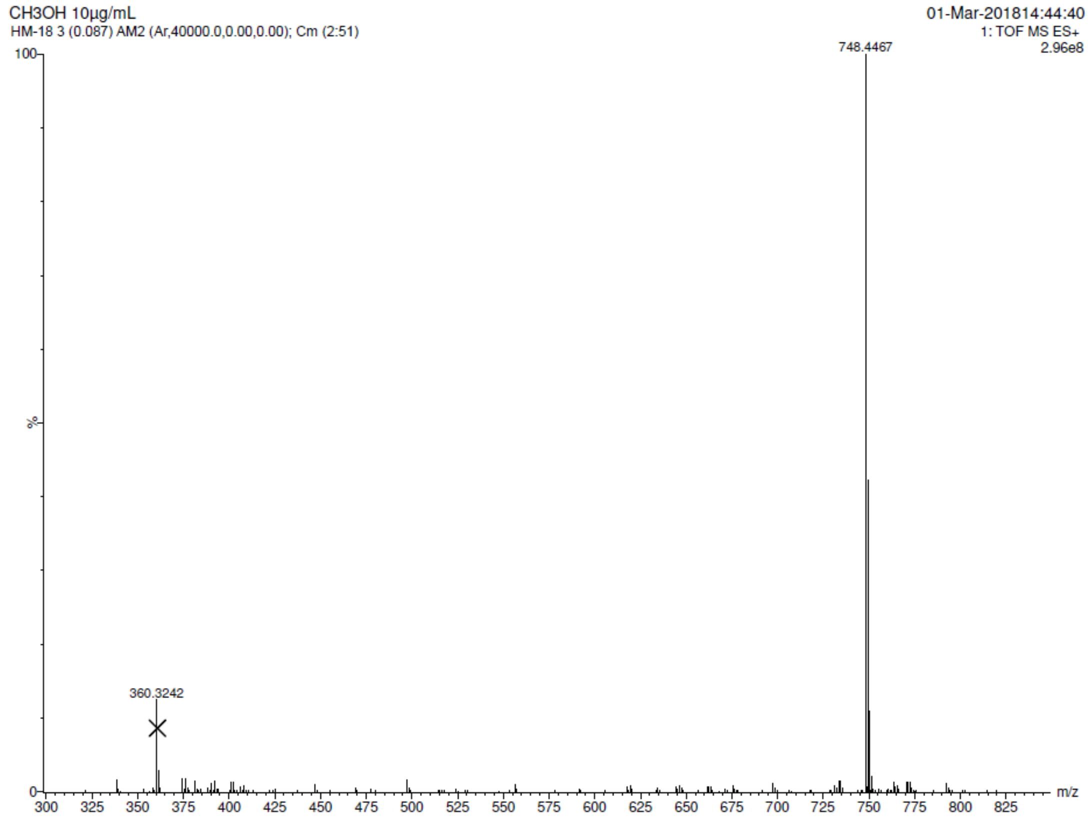

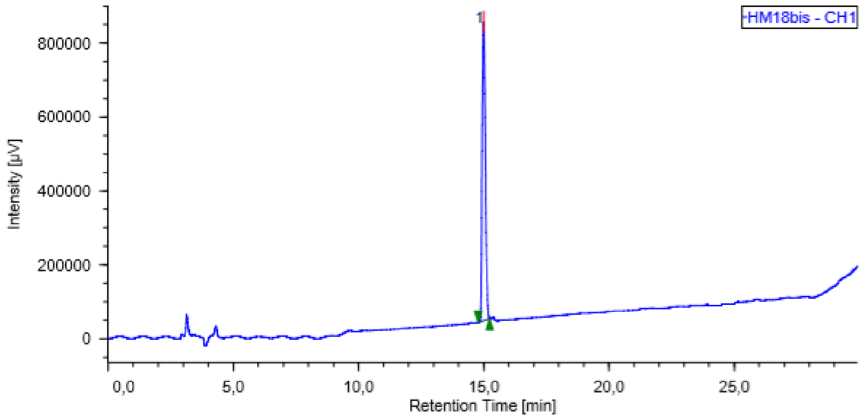

8 (WGY)**:** white powder; yield: 23%; calculated MW: 424.45, MS (ESI) *m/z* = 425.22 ([M+H]$^+$), HRMS ([M-H]$^-$) for $C_{22}H_{24}N_4O_5$: calculated 423.1667, found 423.1668; HPLC: t_r = 15.51 min, 94.82% at 220 nm, Method A.

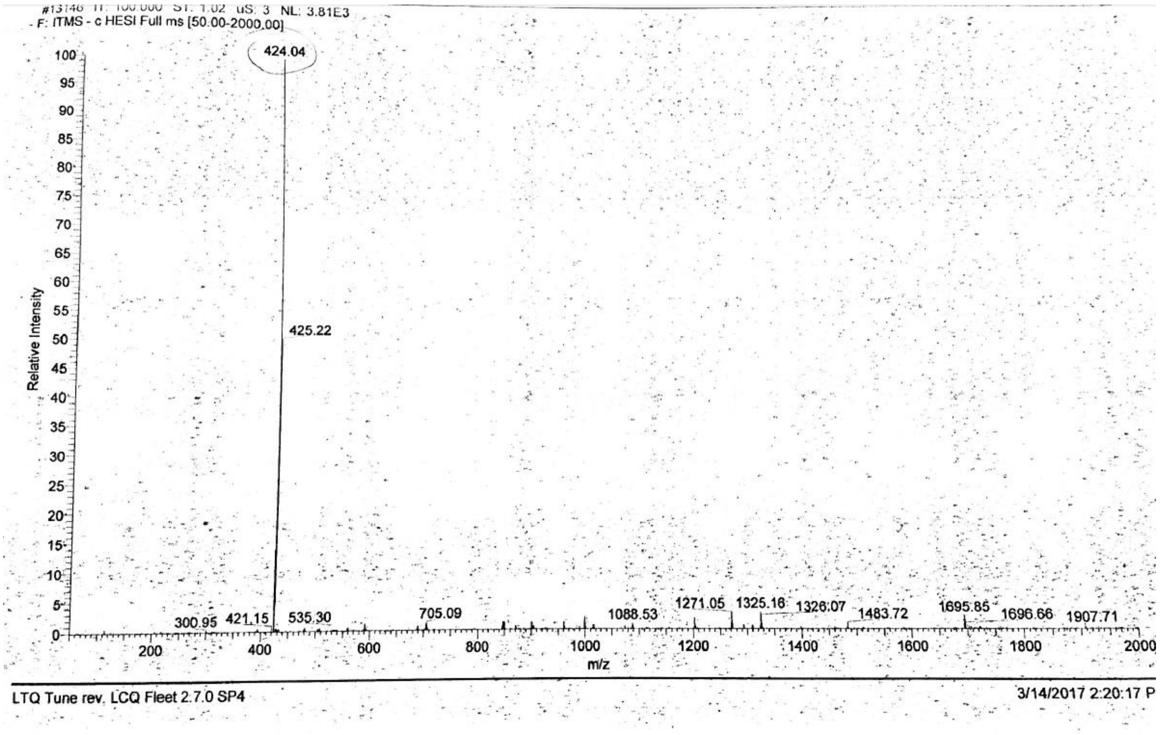

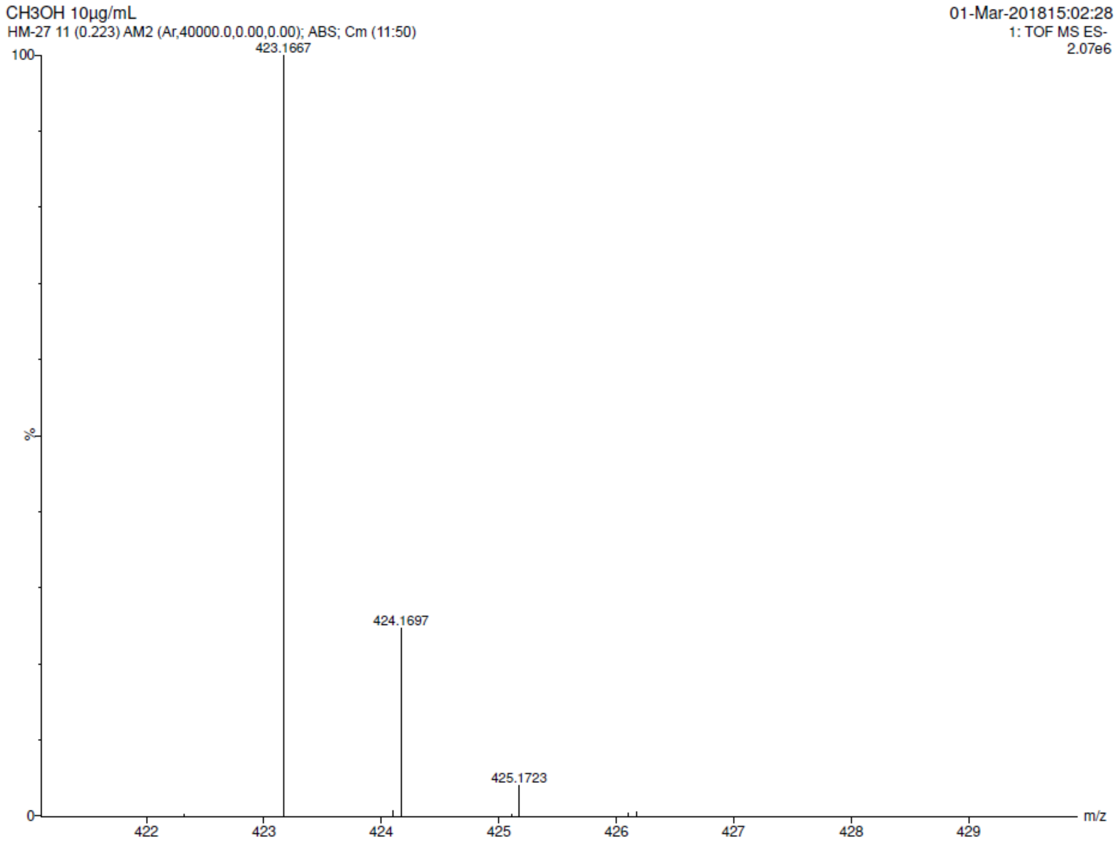

CH3OH 10µg/mL
HM-27 11 (0.223) AM2 (Ar,40000.0,0.00,0.00); ABS; Cm (11:50)
01-Mar-201815:02:28
1: TOF MS ES-
2.07e6
423.1667
424.1697
425.1723
%
m/z

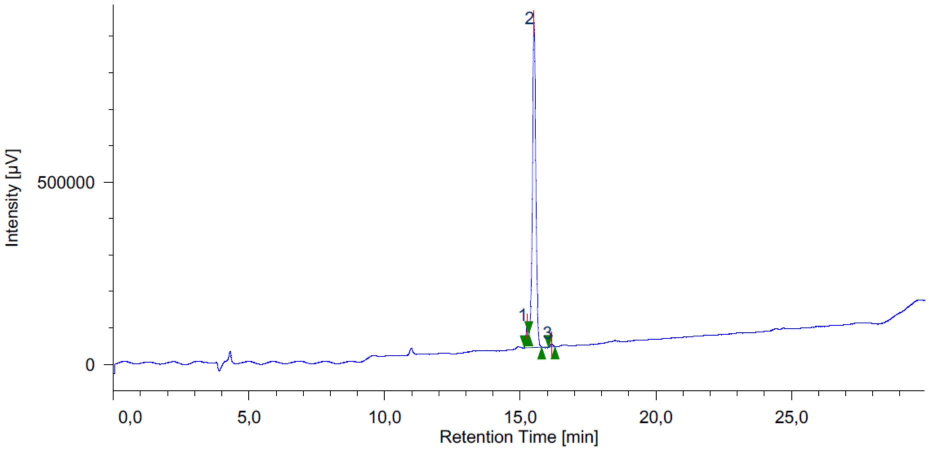

Intensity [µV]
500000
0
0,0
5,0
10,0
15,0
20,0
25,0
Retention Time [min]

9 (YPWF-CONH$_2$)**:** white powder; yield: 26%; calculated MW: 610.70, MS (ESI) *m/z* = 611.3 ([M+H]$^+$); HPLC: tr= 14.36 min, 97.5 % at 280 nm, Method A.

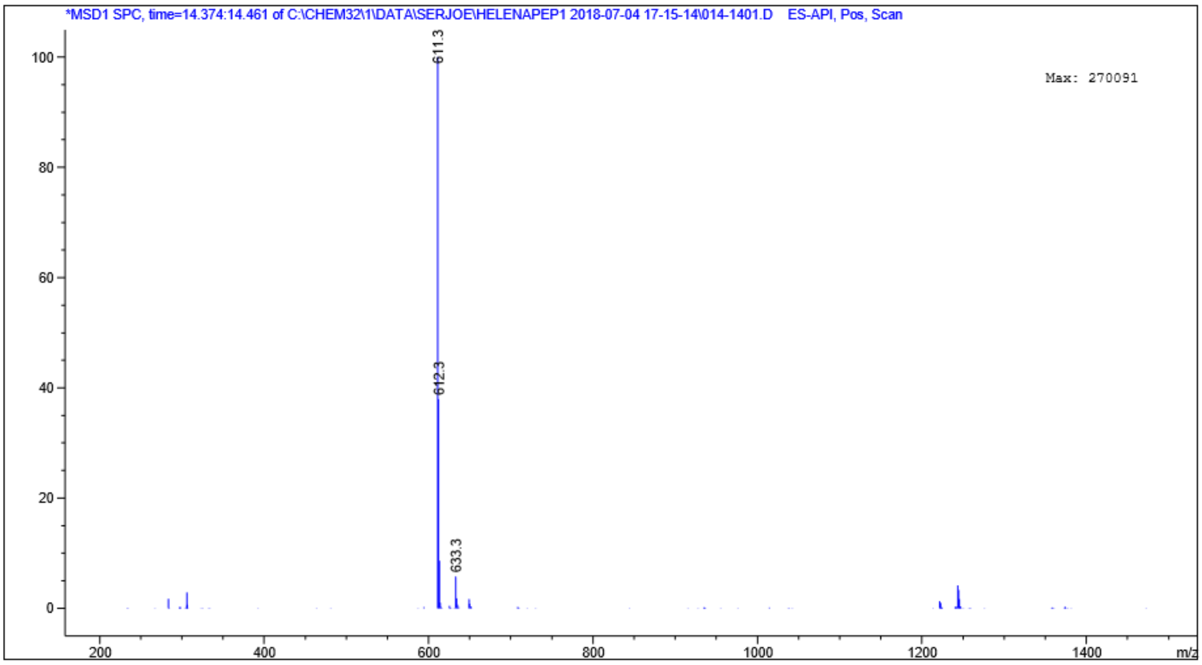

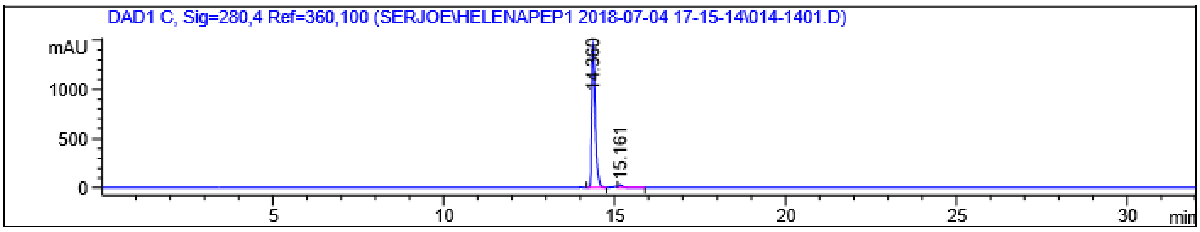

Supplement: Supplementary file 1 — Supplementary Information. [file 41598_2019_56708_MOESM1_ESM.pdf]
